# 2020 International brain–computer interface competition: A review

**DOI:** 10.3389/fnhum.2022.898300

**Published:** 2022-07-22

**Authors:** Ji-Hoon Jeong, Jeong-Hyun Cho, Young-Eun Lee, Seo-Hyun Lee, Gi-Hwan Shin, Young-Seok Kweon, José del R. Millán, Klaus-Robert Müller, Seong-Whan Lee

**Affiliations:** ^1^School of Computer Science, Chungbuk National University, Cheongju, South Korea; ^2^Department of Brain and Cognitive Engineering, Korea University, Seoul, South Korea; ^3^Department of Electrical and Computer Engineering, University of Texas at Austin, Austin, TX, United States; ^4^Machine Learning Group, Department of Computer Science, Berlin Institute of Technology, Berlin, Germany; ^5^Max Planck Institute for Informatics, Saarbrucken, Germany; ^6^Department of Artificial Intelligence, Korea University, Seoul, South Korea

**Keywords:** brain-computer interface (BCI), electroencephalogram, competition, open datasets, neural decoding

## Abstract

The brain-computer interface (BCI) has been investigated as a form of communication tool between the brain and external devices. BCIs have been extended beyond communication and control over the years. The 2020 international BCI competition aimed to provide high-quality neuroscientific data for open access that could be used to evaluate the current degree of technical advances in BCI. Although there are a variety of remaining challenges for future BCI advances, we discuss some of more recent application directions: (i) few-shot EEG learning, (ii) micro-sleep detection (iii) imagined speech decoding, (iv) cross-session classification, and (v) EEG(+ear-EEG) detection in an ambulatory environment. Not only did scientists from the BCI field compete, but scholars with a broad variety of backgrounds and nationalities participated in the competition to address these challenges. Each dataset was prepared and separated into three data that were released to the competitors in the form of training and validation sets followed by a test set. Remarkable BCI advances were identified through the 2020 competition and indicated some trends of interest to BCI researchers.

## 1. Introduction

**Figure 1:**
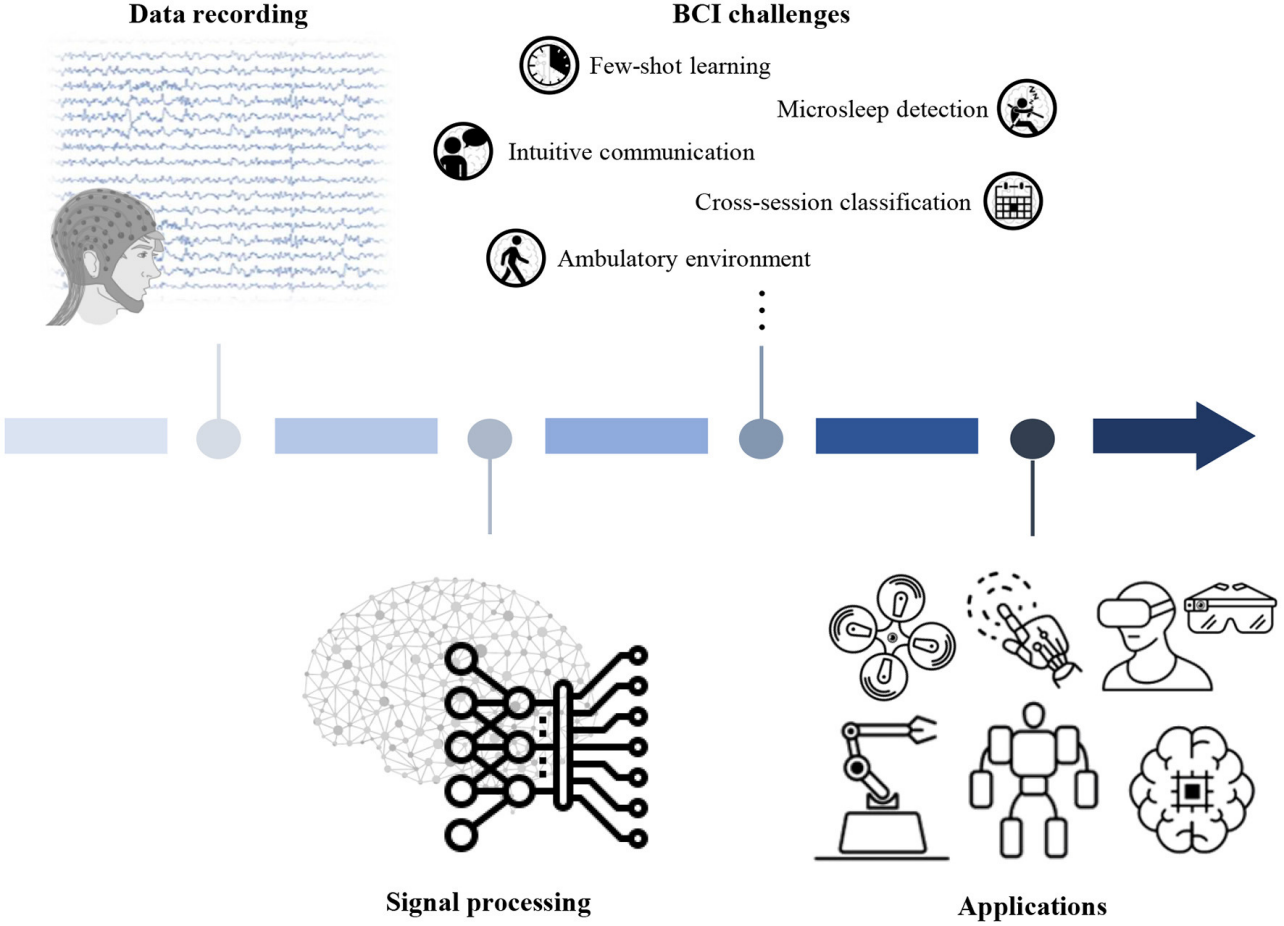
Overview of noninvasive BCI. Every BCI begins its analysis with meaningful feature extraction through signal processing, starting with data recording. Researchers decode EEG data collected from experimental environments suitable for each study using advanced methodologies to develop BCI applications.

This paper presents a review of new frontiers regarding brain-computer interface (BCI) technology and discusses the current BCI technology levels based on the challenges presented in the 2020 international BCI competition. In particular, BCI technology was investigated to provide practical solutions for real-world environments. To achieve this goal, various advanced BCI research topics were summarized into the five categories shown in Figure 1 as a precondition for this review. Beyond simply examining contemporary studies conducted by BCI researchers, we prepared datasets related to the aforementioned challenging topics and released the datasets to the competitors and general public. Various scientists and scholars (98 participants, consisting of 31 teams) participated in the competition to determine the effectiveness of each other's decoding models and methodology used. Through the competition, we were able to confirm the extent of interest and enthusiasm researchers have regarding certain research topics. Hence, we disclosed the datasets and evaluated the models' performances objectively to determine the extent to which current BCI studies have progressed. As a result, we identified current state-of-the-art decoding models and the development direction of non-invasive BCI to obtain a glimpse of how BCI may evolve in the future.

### 1.1. Overview of recent BCI advances

Bridging the communication gap between humans and computers has led to innovative advances using various tools (Millán and Mouriño, 2003; Dornhege et al., 2007; Wolpaw and Wolpaw, 2013; Müller-Putz et al., 2015; Kinney-Lang et al., 2020). BCI is a promising tool for communication between humans and external devices (Vaughan et al., 2003; Wolpaw and Wolpaw, 2007; Blankertz et al., 2010). Traditional BCI technology has been used as a means to support communication with the outside world, mainly for patients with impaired movement due to limb paralysis. Over the past 20 years, experimental studies on BCI have progressed rapidly, with promising results in clinical trials involving groups of motor-impaired patients as well as healthy people (Hochberg et al., 2012; Chaudhary et al., 2016; Penaloza and Nishio, 2018; Jeong et al., 2020c; Mane et al., 2020). BCI technology is characterized by acquiring information generated by brain activity through sensors and interpreting it through technologies such as signal processing and machine learning. BCI systems are implemented by recognizing human intentions through specific patterns of neural signals (Kao et al., 2015; Pearson, 2019) using modern signal processing and machine learning (Pascual-Marqui, 2002; Delorme and Makeig, 2004; Blankertz et al., 2008, 2011, 2016; Lemm et al., 2011; Lee M. H. et al., 2019). In particular, recent trends in the development of decoding models have achieved meaningful performance in BCI systems by also applying technologies such as deep learning along with more traditional signal processing methods and machine learning. BCI forms an integrated interface involving hardware and software that can directly decode human intentions for a variety of applications (Dornhege et al., 2007; Müller et al., 2008; Millán et al., 2010; Yadav et al., 2020), as shown in Figure 1.

BCI research has progressed in two forms: invasive and noninvasive (Chaudhary et al., 2016). These forms enable the recording of different types of brain signals. The invasive type involves surgical implantation of micro-sensors in the cerebral cortex and could be used to control neuroprosthetics (Hochberg et al., 2012; Micera, 2017). General invasive modalities, for instance, have been reported as electrocorticography (ECoG) and intracortical neuron recording (INR). These techniques can obtain high quality patterns from high-resolution temporal signals, including the activity patterns of neurons (Nason et al., 2020). However, short usability arises owing to the high possibility of tissue rupture and scar build-up over the passage of time, in the case of INR. In addition, installation through surgical approaches remains unchanged even if using ECoG (Chaudhary et al., 2016; Yadav et al., 2020).

In contrast, despite poor signal quality and low spatial resolution, noninvasive BCI typically remains the preferred method because surgical operations are not required. Noninvasive modalities exist in various forms for the detection of brain signals over the scalp, such as electroencephalography (EEG), magnetoencephalography (MEG), and functional-near-infrared spectroscopy (fNIRS) (Fazli et al., 2012; Ahn et al., 2013; Dähne et al., 2015; Aghajani et al., 2017). EEG is the most widely used modality and can record electrical activity by synaptic excitation of neuronal dendrites within the brain (Rashid et al., 2020). It can detect a variety of control signals, including slow cortical potential (SCP) (Birbaumer et al., 1999; Kübler et al., 2001; Shakeel et al., 2015; Jeong et al., 2020b), event-related potential (ERP) (Kübler et al., 2009; Riccio et al., 2013; Won et al., 2017), steady-state visual evoked potential (SSVEP) (Müller-Putz and Pfurtscheller, 2007; Lesenfants et al., 2014; Kwak et al., 2017; Zheng et al., 2020), error-related potential (ErrP) (Blankertz et al., 2003, 2011; Buttfield et al., 2006; Chavarriaga and Millán, 2010), and motor imagery (MI) (Blankertz et al., 2008; Suk and Lee, 2011; Ang and Guan, 2016; Leeuwis et al., 2021). As a result, neural decoding has been developed for healthy people and patients along with various applications (Birbaumer et al., 1999; Millán et al., 2004; Pires et al., 2011; Höhne et al., 2014; Abiri et al., 2019).

BCI applications have been investigated for rehabilitating patients and to communicate with external devices such as wheelchairs (Kim et al., 2016; Degenhart et al., 2020), robots (Penaloza and Nishio, 2018; Edelman et al., 2019), and spellers (Kübler et al., 2009; Chen et al., 2015). Furthermore, one of the important achievements of utilizing EEG-based BCI is its application in routine areas of our daily lives [e.g., sleep Cox and Fell, 2020, augmented/virtual reality (AR/VR) Schwarz et al., 2020b; Woo et al., 2021, emotion recognition Zhang et al., 2019; Kim et al., 2020, biometrics Maiorana, 2020, and environmental control Zhuang et al., 2020] that have arisen through advances in various studies, such as the development of BCI hardware, neurophysiological knowledge, and machine learning. In this review, we mainly focus on the advances in machine learning.

### 1.2. BCI challenges

Despite advances in decoding skills, current BCI systems still face significant technical issues (Yadav et al., 2020). To be widely commercialized such as in other research fields (e.g., speech recognition and computer vision), the performance of the neural interface must be stable and robust under different conditions. Recent BCI advances have been reported alongside trends in technical approaches. In this review, we primarily focus on the significant and substantial challenges associated with BCI software. These challenges were presented to help overcome the limitations and drawbacks of current BCIs. Regarding the commercialization and generalization of practical BCIs, five technical challenges and our datasets were defined as follows:

*i*) Data Set-A: Few-shot EEG learning for short-calibration
*ii*) Data Set-B: Micro-sleep detection from a single channel
*iii*) Data Set-C: Imagined speech decoding for intuitive BCI communication
*iv*) Data Set-D: Cross-session classification of upper-limb movements
*v*) Data Set-E: EEG(+ear-EEG)-based ERP detection under ambulatory environment

### 1.3. Relevance of BCI competition

For decades, BCI research has progressed in many areas and has gained popularity. However, the technology is usually expensive, complex to operate, and requires a long set-up time, making it difficult to collect data. Owing to these difficulties, reliable EEG data collected for BCI competitions I–IV (Sajda et al., 2003; Blankertz et al., 2004, 2006; Tangermann et al., 2012) in the past continue to be used in many studies. Therefore, we decided to provide new-frontier datasets through the 2020 international BCI competition to many investigators to better evaluate BCI advances and the current level of BCI technology. This review is based on a wide investigation and the insights gained from organizing the international BCI Competition (website: https://osf.io/pq7vb/; DOI: https://doi.org/10.17605/OSF.IO/PQ7VB), which was jointly held with the 9^*th*^ IEEE Winter Conference on Brain-Computer Interface (http://brain.korea.ac.kr/bci2021/competition.php). The competition was an online-based and comparably large event whose purpose was to evaluate the latest state-of-the-art BCIs and their respective performances.

We outline the latest methodologies and directions of research in each field. We list descriptions of the datasets utilized during the competition and describe the performances demonstrated by competition participants. Consequently, the released datasets are considered challenging and point to remaining issues in this discipline that if solved will allow BCI technology to become more closely integrated in daily life.

### 1.4. Necessity of open datasets for BCI

As mentioned earlier, BCI is quite difficult to commercialize owing to limitations such as a lack of data and the absence of affordable measuring equipment (Jeong et al., 2020a). In addition, it is a challenging topic in related research communities because it consists of high-dimensional data containing large amounts of noise caused by *in vivo* interference as well as the surrounding environment. To solve these problems, high-quality (open) data measured through various experiments are essential. In this regard, many universities and research institutes contribute to the advancement of technology by providing BCI data or holding competitions (Kaya et al., 2018; Shin et al., 2018; Lee M. H. et al., 2019; Jeong et al., 2020a; Stieger J. R. et al., 2021). As such, open datasets are considered an important asset for promoting BCI research and practices. By providing the data for this competition, scientists and researchers from various fields such as signal processing, data analytics, and artificial intelligence should also be able to develop new algorithms, methods, and applications leading to better commercialization of BCI technology.

## 2. Overview of the competition datasets

For the competition, BCI datasets were prepared according to the challenging issues discussed previously. Five competition datasets were separately prepared and consisted of training, validation, and test sets. Participants were able to train their models with the training sets and measure their BCI performance using the validation sets. Lastly, a test set was used to evaluate the final version of the models developed by the participants based on decoding performance. BCI studies have developed models and compared performance based on locally obtained data or limited public datasets. Through the 2020 international competition, we expect that the released datasets may contribute to the creation of advanced decoding models and can be used for fair performance comparisons. Using competition datasets to compare and evaluate various researchers' BCI decoding performance has not been easily achieved in BCI studies. However, this type of comparison can identify the level and direction of the development of BCIs by study groups using common comparators.

All experimental protocols and settings that we collected were reviewed and approved by the ethical committee of the Institutional Review Board (IRB) at Korea University. The IRB information for the data is as follows: 1040548-KU-IRB-16-159-A-2 (Data Set-A); 1040548-KU-IRB-17-46-A-2 (Data Set-B, only validation and test sets); KUIRB-2019-0143-01 (Data Set-C); 1040548-KU-IRB-17-172-A-2 (Data Set-D); KUIRB-2019-0194-01 (Data Set-E). After ensuring that subjects understood the experiments and provided their written consent according to the Declaration of Helsinki, they signed a form that agreed to the anonymous public release of their data.

### 2.1. Data Set-A

#### 2.1.1. Background

Short calibration, which uses minimal training data in most BCI paradigms that aim to develop practical systems, is a major challenge (Benaroch et al., 2021; Ko et al., 2021a). The development of decoding models using less training data, described as few-shot learning, which allows a model to learn a method that enables fast adaptation to a new task or environment (Hospedales et al., 2020), is one of the challenges associated with machine learning and deep learning (An et al., 2020). For few-shot learning, recent studies have been conducted that mostly used only 10 samples from datasets to estimate the true labels for an entire dataset (Supplementary Table 1). With this 10-shot classification, Azab et al. (2019) proposed a weighted logistic regression-based transfer learning algorithm that achieved an accuracy of 71.0–75.6%. McCartney et al. (2019) proposed a zero-shot EEG-to-image brain-decoding approach and achieved classification accuracies of 61.3 and 62.2% for zero-calibration. An et al. adopted a relation network to efficiently learn class representative features among multiple subjects. They evaluated the proposed network in 5-, 10-, and 20-shot settings per class. The results achieved averaged accuracies of 71.0% (±10.5), 72.6% (±11.7), and 74.6% (±10.2), respectively (An et al., 2020).

#### 2.1.2. Challenge

Classification of MI data is a significant challenge due to the lack of data volume, inter-subject variability, low signal-to-noise ratio, and complex dynamics of MI. The purpose of Data Set-A was to classify the MI data of a certain subject using minimal training data based on few-shot learning. Few-shot learning aims to train generalized classifiers using very small amounts of training data, contrary to the normal practice of using a large amount of data. The expected baseline of this part of the competition was to achieve high classification performance using only the data provided after a short-calibration session to develop BCI practical systems. For example, if BCIs are to be used by people, it is important to build a highly accurate system, but at the same time, the models should be learned quickly, and people should be able to use them without excessive recalibration (Fazli et al., 2009; Kindermans et al., 2014; Ko et al., 2021a). Consequently, the relevant data were released to develop technologies that enabled the required minimization calibration as a condition for implementing a practical BCI by the participants.

#### 2.1.3. Experimental protocols and paradigm

**Figure 2:**
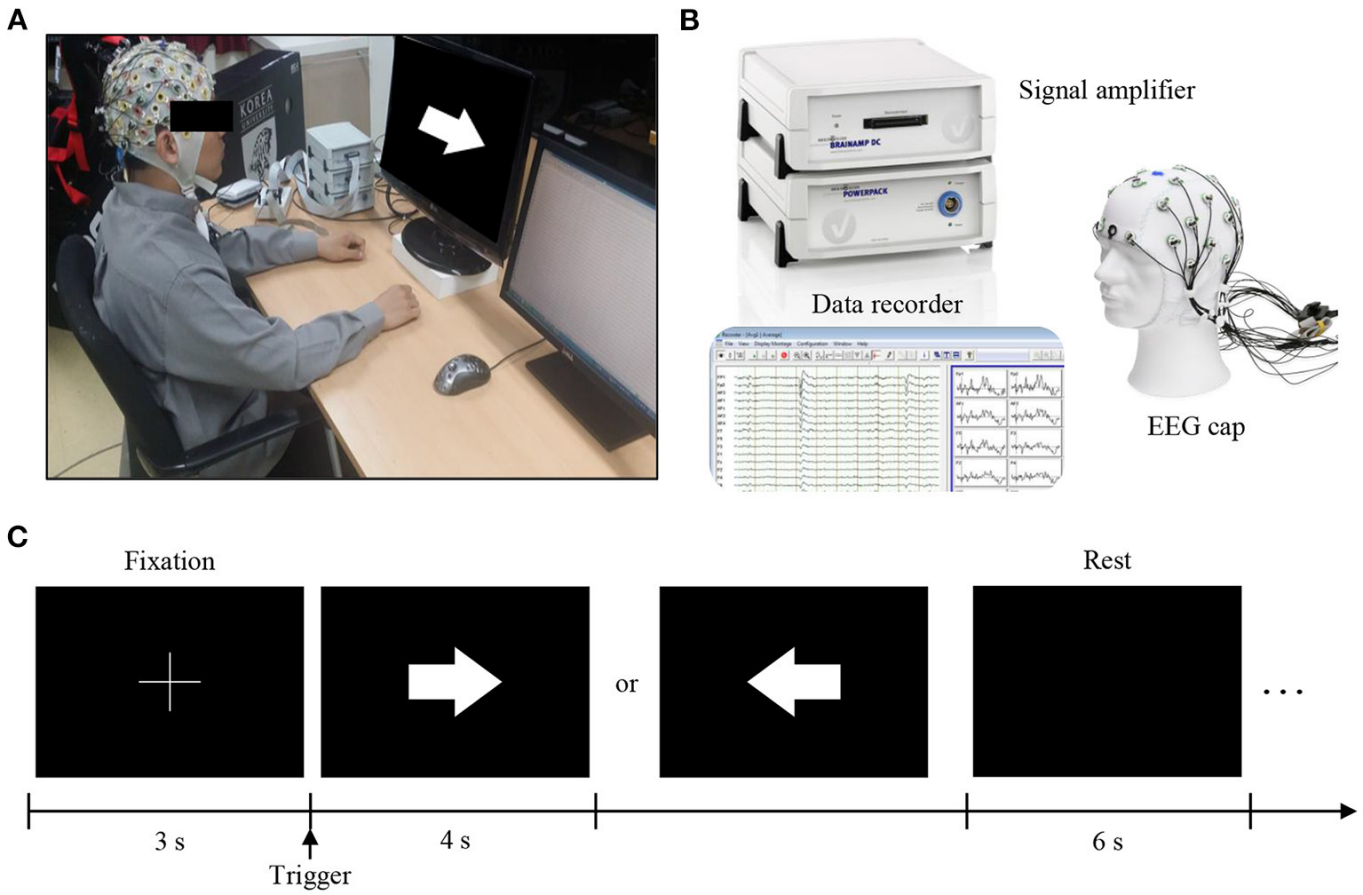
Experimental setup and protocol. **(A)** During the experiment, the subjects were seated comfortably in a chair with armrests at 60 (±5) cm in front of a 21-inch LCD monitor. **(B)** EEG signals were measured using brainwave collection equipment (BrainAmp, BrainProducts GmbH, Germany) and data recorders (BrainVision, BrainProducts GmbH, Germany). **(C)** In the experimental paradigm, for all blocks, the first 3 s of each trial began with a fixation cross that appeared at the center of the monitor to prepare subjects for the MI task. Afterward, the subject performed the imagery task using the appropriate hand for 4 s when a right or left arrow appeared as a visual cue. After each task, the screen remained blank for 6 s. The given data set provides only 4 s MI tasks.

EEG data for two motor imagery tasks were recorded during a single session across 20 subjects. During a session of the experiment, the subjects were seated comfortably in a chair with armrests at 60 (±5) cm in front of a 21-inch LCD monitor (Figure 2A). The approximate horizontal and vertical visual angles were 37.7 and 28.1°, respectively. During the experiment, subjects were asked to relax their muscles and minimize their eye and muscle movements.

EEG signals were recorded at a sampling rate of 1,000 Hz and collected using 62 Ag/AgCl electrodes (nasion-referenced and AFz-grounded). The EEG amplifier used in the experiment was a BrainAmp (Brain Products GmbH, Munich, Germany) shown in Figure 2B. The impedance was kept 10 kΩ below during the experiment.

The MI paradigm was designed according to a well-established protocol (Figure 2C). For all blocks, the first 3 s of each trial began with a black fixation cross that appeared at the center of the monitor to prepare subjects for the MI task. Afterward, each subject performed an imagery grasping task with the appropriate hand for 4 s once a right or left arrow appeared as a visual cue. After each task, the screen remained blank for 6 s. The given dataset provided only 4 s MI tasks. The data disclosed to the participants for the competition was prepared by ourselves and was reviewed by the Korea University IRB.

#### 2.1.4. Data configuration

The data were configured as training (10 shots), validation (10 shots), and test (10 shots) sets according to each class, providing a total of 20 shots (10 shots×2 classes) for each stage. Only the training and validation sets were provided to the competitors in order to obtain a fair evaluation. A description of the data is provided in Supplementary Table 2.

#### 2.1.5. Competition outcomes

**Figure 3:**
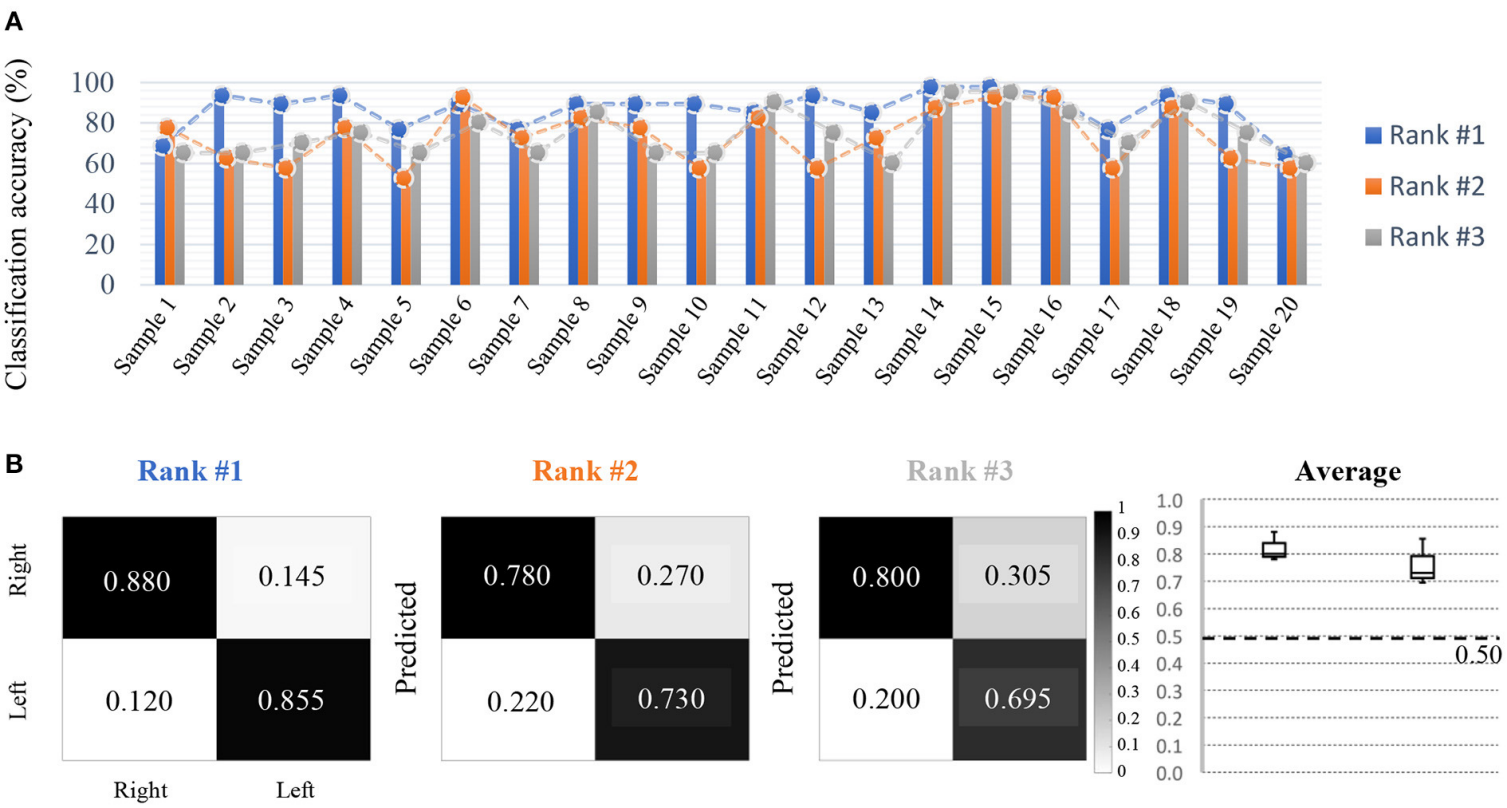
Competition outcomes. **(A)** Decoding results on short-calibration BCI (Accuracy), where the difference between the top performer and the other two performers are significant, but the difference between the second and third places is not significant. All participants demonstrate consistent decoding performance regarding the samples used. For short-calibration BCI, both right-hand MI and left-hand MI classes were used. **(B)** Despite using a small amount of data, on average, the true positive rate and performance were approximately 0.200 (20%) higher than the chance rate.

The results of the participants' analyses are depicted in Figures 3A,B. Overall, participants achieved classification results over the chance rate accuracy in 2-way, 10-shot settings. The top-scoring participants showed a statistically significant increase in performance compared to other participants through the paired *t*-test (*p* < 0.01). Interestingly, although the final averaged performances across all samples were similar, the performance differed for each subject sample according to each participant's model (Figure 3A). Therefore, the dataset was demonstrated to not be biased according to each subject sample. Furthermore, the performance of the models developed was generally stable, producing high performance utilizing minimized training data, and also exhibited reliable true positive rates for each class, despite using only a few data samples (Figure 3B). This allows us to state that for short calibration, the stability of the model requires not only an improvement in average performance across all subject samples, but also stable performance per class.

#### 2.1.6. Contribution

Through the competition using Data Set-A, the state-of-the-art performance for few-shot EEG learning was examined. Despite the 2-way, 10-shot setting, the results achieved surpassed the chance-level accuracy across all samples. A variety of approaches have been reported to solve the short calibration problem in BCI, such as adapting transfer learning methods and applying data augmentation algorithms. The few(zero)-shot learning approach is expected to be the most complex and difficult approach because it addresses the scenario where a classifier must adapt to accommodate classes that are not seen during training when only a few labeled samples per class are provided (An et al., 2020). In addition, unlike signals from other domains, the inconsistent features of an EEG can reveal different information for each shot (trial), which may make it difficult to train the model. In this respect, through the competition, when first trying few(zero)-shot learning for short-calibration BCI, we recommend adopting explicit few-shot learning algorithms while confirming the quality of the signals for each trial as well as attempting to reflect invariability from temporal, spatial, or spectral information.

### 2.2. Data Set-B

#### 2.2.1. Background

**Figure 4:**
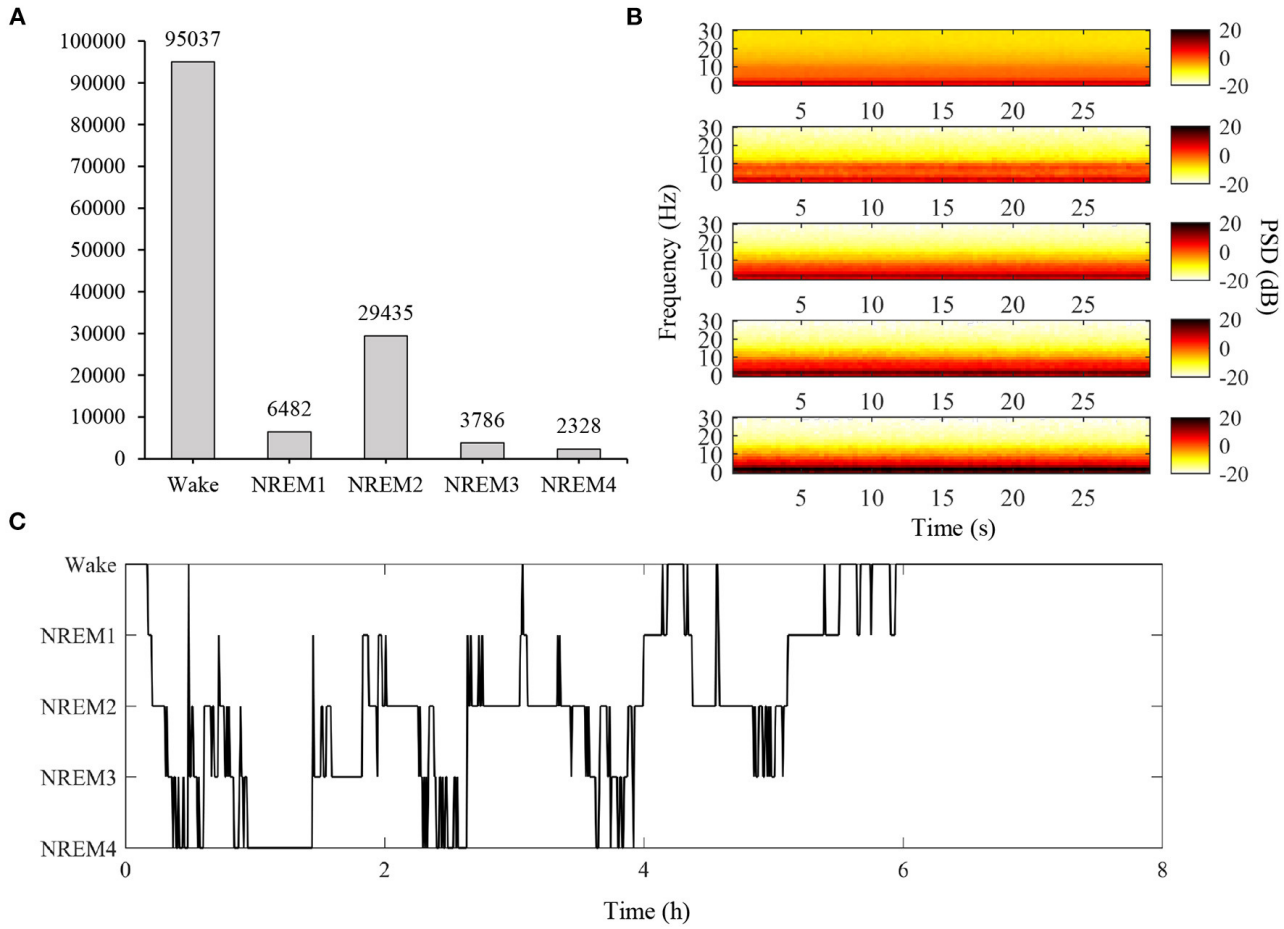
Overview of training data from modified Sleep-EDF dataset. In Data Set-B, an open dataset was modified to provide large sleep stage data, unlike other competition tracks. **(A)** Total number of samples according to classes in the training set. For this competition, we selected a total of 50 EEG data from 20 males and 30 females, excluding missing data and subjects outside the range of 25–56 years of age. **(B)** Spectrogram of DataSample01 based on classes. From top to bottom, the figure represents the average spectrogram for Wake, NREM1, NREM2, NREM3, and NREM4 states (PSD: power spectral density). **(C)** Hypnogram of DataSample01. (PSD, power spectral density).

Consciousness is an outcome of the neuronal network in the brain (Dehaene and Changeux, 2004; Tononi, 2004; Koch et al., 2016; Lee et al., 2017). To recognize the consciousness of people, BCI technology has received attention in the medical and engineering fields. Sleep stage classification is a BCI task associated with recognizing consciousness (Laureys et al., 2004; Patil et al., 2007; Jordan et al., 2008; Boly et al., 2009; Rosipal et al., 2013; Koch et al., 2016). There are two consciousness states during sleep: rapid eye movement (REM) and non-rapid eye movement (NREM) sleep. Classifying these states is important for the diagnosis and treatment of patients with sleep disorders (Lee M. et al., 2019; Perslev et al., 2019). Therefore, a large-scale EEG database during night sleep, Sleep-EDF, was previously published (Kemp et al., 2000) (Figure 4). In contrast, various BCI tasks involve recognizing a user's consciousness during driving: fatigue estimation, drowsiness detection, and micro-sleep detection. To investigate the conscious state, various methods have been used, such as the Karolinska sleepiness scale (KSS) and percentage of eye closure (PEREC) (Zhou F. et al., 2020). Gao et al. (2019). evaluated driver fatigue using the KSS, and estimated fatigue from EEGs using an EEG-based spatial-temporal convolutional neural network achieving 0.93 accuracy. Ko et al. (2021b) categorized drowsiness using a percentage of eyelid closure (PERCLOS) levels, and they classified drowsiness using a multi-scale neural network (Supplementary Table 3).

#### 2.2.2. Challenge

Micro-sleep is a brief episode within the sleep cycle, which can last from 1 to 15 s. These episodes of micro-sleep occur most frequently when a sleepy person is trying to fight off sleep and remain awake, especially in the driving environment (Skorucak et al., 2020b). This is a critical problem that can result in severe accidents (Williamson and Feyer, 2000). Despite the importance of micro-sleep during driving, the development of micro-sleep detection has been delayed because of too many variations in associated experiments, such as whether the study involves a simulation or real driving (Gao et al., 2019; Ko et al., 2021b), use of different physiological signals (Gao et al., 2019; Karuppusamy and Kang, 2020), and the criteria used to define micro-sleep (Gao et al., 2019; Ko et al., 2021b). On the one hand, micro-sleep is similar to NREM stage 1 during night sleep because NREM stage 1 is the transition period between wakefulness and sleep (Balaji et al., 2021). In micro-sleep, EEG theta activity is dominant like NREM stage 1 (Wu et al., 2013; Skorucak et al., 2020a). We suggest that using a large-scale EEG database during night sleep can improve micro-sleep detection performance, regardless of variations among experiments. Another important issue is the single-channel classification. For real-life applications, too many EEG channels interrupt driving and reduce comfort. However, reduction of EEG channels causes decrements in BCI performance (Lim et al., 2014). Thus, maintaining performance is a critical issue, especially when EEG channels are reduced. We hoped that participants detected micro-sleep using night sleep with only 2 channels. This would accelerate the development of micro-sleep detection in various fields.

#### 2.2.3. Experimental protocols and paradigm

Training: We used the sleep-EDF database that includes 197 polysomnographic sleep recordings during the whole night, containing EEG, EEG, electroculography (EOG), chin electromyography (EMG), and event markers to analyze sleep patterns. The 153 sleep cassette files were obtained in a 1987–1991 study of age effects on sleep in healthy Caucasians who do not have any sleep-related medications and aged 25–101. The sampling frequency of EEG signals were 100 Hz. Sleep-EDF is presented in detail at https://physionet.org/content/sleep-edfx/1.0.0/.

**Figure 5:**
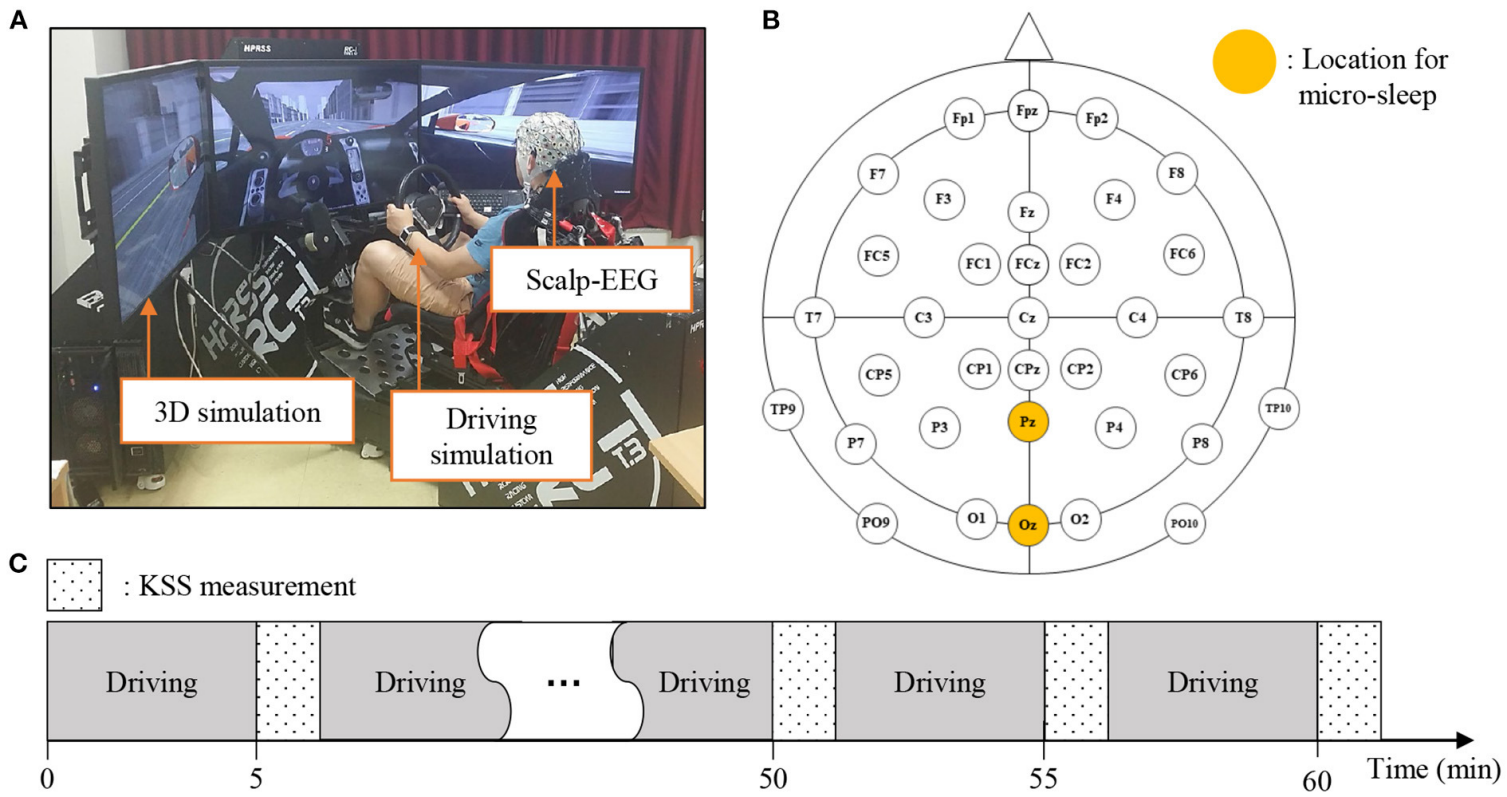
Experimental setup and protocol corresponding to validation and test data. **(A)** Provides an experimental environment that simulates 3D driving while wearing the EEG cap. **(B)** Locations of electrodes for recording EEG. Pz and Oz were used to detect micro-sleep. **(C)** The experiment was conducted for approximately 1 h, and the subjects were evaluated based on the Karolinska sleepiness scale (KSS) 13 times, which was used to indicate their drowsiness level while driving.

Validation and Test: Ten subjects participated in this experiment. The driving simulator was composed of three 42-inch-wide monitors and driving control tools, such as an accelerator, a brake pedal, a steering wheel, and a seat (Figure 5A). The simulation was developed using the Unity 3D engine software (http://unity3d.com). Subjects were seated with fastened seat belts to provide a real-world driving simulation. The EEG signals used the BrainAmp amplifier (Brain Products GmbH, Munich, Germany), and the sampling frequency was set to 100 Hz. The experiment was conducted with the impedance of all electrodes below 10 kΩ. We selected the Pz and Oz channels from Sleep-EDF (Figure 5B). The experiment was conducted for approximately 1 h, and the subjects were evaluated using the Karolinska sleepiness scale (KSS) 13 times, which represents the drowsiness scale from 1 to 9 (Figure 5C).

#### 2.2.4. Data configuration

Training: We created a training set by converting the Sleep-EDF dataset. A total of 50 EEG data (from 20 males and 30 females) were selected, excluding missing data and subjects outside the range of 25–56 years of age. Following standard rules, sleep stages (W, R, 1, 2, 3, 4, and M) were assigned for each 30 s window. A detailed description of the data is provided in Supplementary Table 4.

Validation and Test: It is a dataset for detecting micro-sleep using night sleep (training set) containing information regarding EEG sleep patterns. The data are divided into 2-class samples indicating micro-sleep when KSS ≥ 7 and wakefulness when KSS ≤ 6 for each 30 s window. A detailed description of the data is provided in Supplementary Table 4.

#### 2.2.5. Competition outcomes

**Figure 6:**
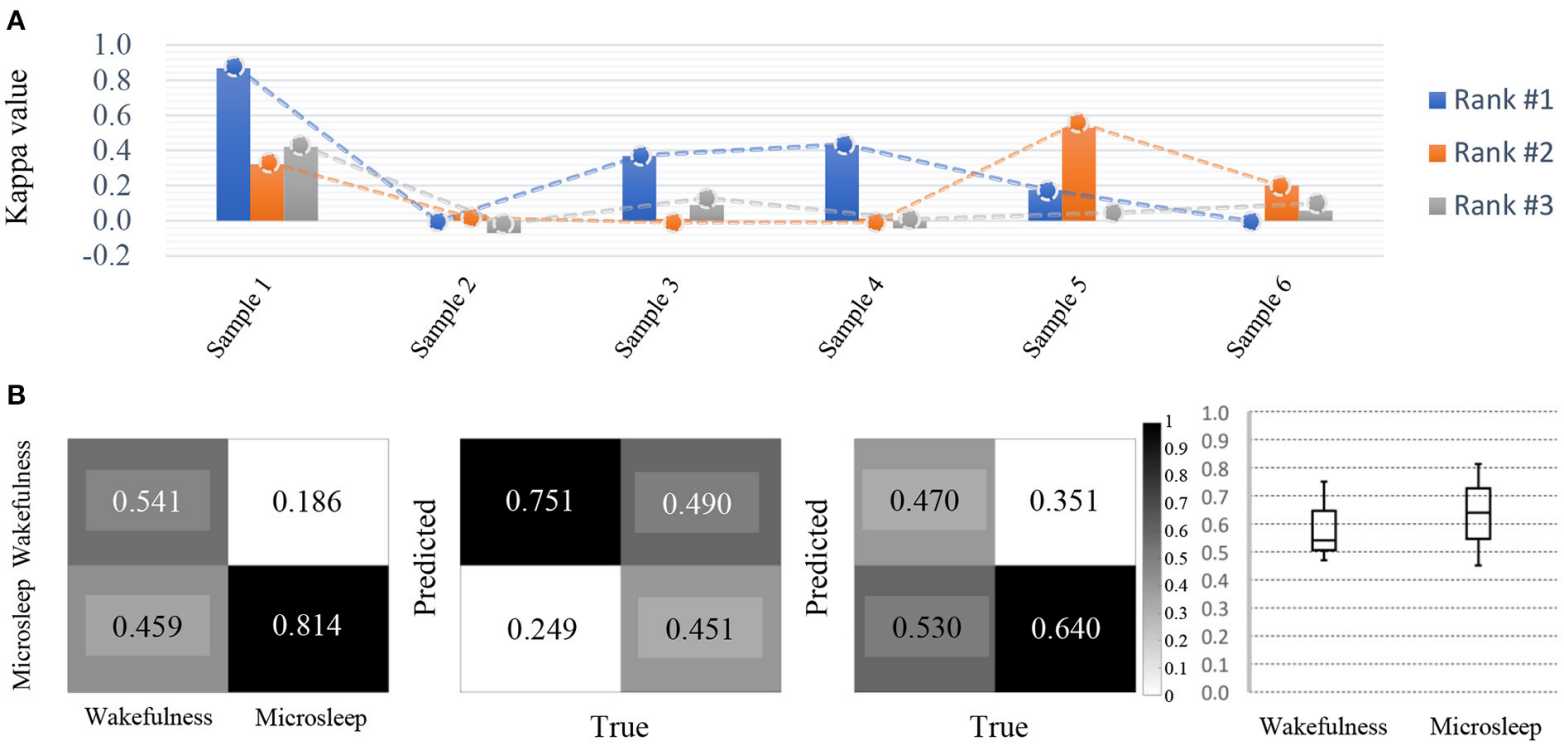
Competition outcomes. **(A)** Micro-sleep detection results (kappa value) and achieved decoding performance indicating large deviations among participants. All participants show relatively large decoding performance deviations for each sample. **(B)** For micro-sleep detection, despite being the top-ranked models, detection accuracy is still insufficient and there is a large deviation in the performance achieved by each participant (based on Cohen's kappa value).

The performance evaluation for micro-sleep detection was based on Cohen's kappa value because of data imbalance (Figure 6A). The average kappa value recorded by the model achieved the highest performance was 0.308, and the second-best performance was 0.177, which indicated a relatively large deviation between performances. Therefore, it can be inferred that there may be many effective solutions using this type of data, but the problem is not easy to solve given the variations in performance achieved by the participants. Therefore, creating a classification model that can accurately explore these areas is at the core of a well-performing conservative BCI decoding model.

In addition, based on the confusion matrix, micro-sleep using brain signals is difficult to detect accurately between classes (Figure 6B). This result was due to how different models learned the environment from the training set as well as the evaluation and test sets. As a result, use of even the same micro-sleep data suggests that model learning will also vary depending on the subject's current environment.

#### 2.2.6. Contribution

The competition results using Data Set-B showed that it may be possible to detect micro-sleep using large-scale EEG data based on night sleep. We had hoped that this approach would help industries to achieve their goals without incurring high data collection costs. However, the low performance achieved by the competitors is still an issue and needs to be improved. Because only a single channel was used, they achieved a relatively lower performance than results reported in previous literature, where multiple channels were used. The competition highlighted the potential of large-scale EEG data to detect micro-sleep but also confirmed the significant difficulties associated with single-channel EEG analysis. Further research could consider this novel approach to recognize consciousness using large-scale EEG data during night sleep.

### 2.3. Data Set-C

#### 2.3.1. Background

People hope for a future in which BCI decodes what one intuitively imagines and outputs it to the real-world environment (Lee et al., 2019a, 2020a). Once the imagined word or conversation is decoded by the BCI system, it can then be used as a neural command to output user-imagined words through speech synthesis or to control robots and devices based on words (Herff et al., 2017; Moses et al., 2021). Consequently, the effectiveness and utility of translating imagined speech are significant issues. To implement these types of BCI, relevant features of the imagined speech paradigm were investigated, aiming to improve the effectiveness of capturing speech-related brain activity (Lotte et al., 2015; Brumberg et al., 2016). Recently, researchers have investigated a variety of methods, especially deep learning, which has evolved along with natural language processing techniques, to precisely capture phoneme-level speech from brain signals (Herff et al., 2015; Anumanchipalli et al., 2019; Makin et al., 2020). In Supplementary Table 5, the relevant studies to date on intuitive imagery analysis are summarized. This allowed us to infer what trends and directions are being used to implement intuitive BCI communication. With the aim of decoding intuitive speech, BCI is evolving in conjunction with analyses using various paradigms (Cooney et al., 2018; Lee et al., 2020b; Lee S. H. et al., 2021).

#### 2.3.2. Challenge

Imagined speech can be a key paradigm toward developing intuitive systems that users can easily operate (Lee et al., 2019a). Recognizing the user's intuitive imagery and translating it to the outside world is one of the critical functions of BCIs. Using an imagined speech paradigm, communication using a BCI could significantly improve because it could directly convey the user's intention through the imagined speech or word itself instead of through the spelling of individual letters (Pei et al., 2011; Herff et al., 2017; Lee et al., 2020a). Simultaneously, this technology could apply this interpreted intuitive imagery to control external devices. Imagined speech is an emerging paradigm that can transfer a user's intention to external devices (Tankus et al., 2012; Nguyen et al., 2017; Tian et al., 2018; Lee et al., 2020a). An imagined speech paradigm could provide crucial advantages compared to conventional BCI paradigms (e.g., MI). For example, enlarging the number of classes in MI relies on the movement of body parts, which may naturally overlap when many classes are necessary (Herff et al., 2017; Lee et al., 2020a). Conversely, speech attributes of different classes may allow more variations between classes without overlapping concepts. Moreover, intuitive decoding directly matches the interaction between user intentions and device feedback in real-world environments (Moses et al., 2019). Eventually, this characteristic of the intuitive paradigm could contribute to the development of practical BCI systems that provide a high degree of freedom to the user (Herff et al., 2015; Lee et al., 2019b). Hence, BCI favors a technique that decodes human intuitive imagery, and datasets were prepared with respect to imagery speech paradigms to evaluate current models through the competition. However, compared to the conventional BCI paradigms (such as MI or ERP), the multiclass classification performance of imagined speech is remaining at a relatively inferior level (Lee et al., 2019a). An effective feature selection or classification method for imagined speech may contribute to improving the decoding performance (Lee et al., 2020a). Therefore, we expected through this competition to improve the multiclass classification performance of imagined speech to the level of conventional BCI paradigms, thus enabling simple communication or control of external environments *via* internal speech.

#### 2.3.3. Experimental protocols and paradigm

**Figure 7:**
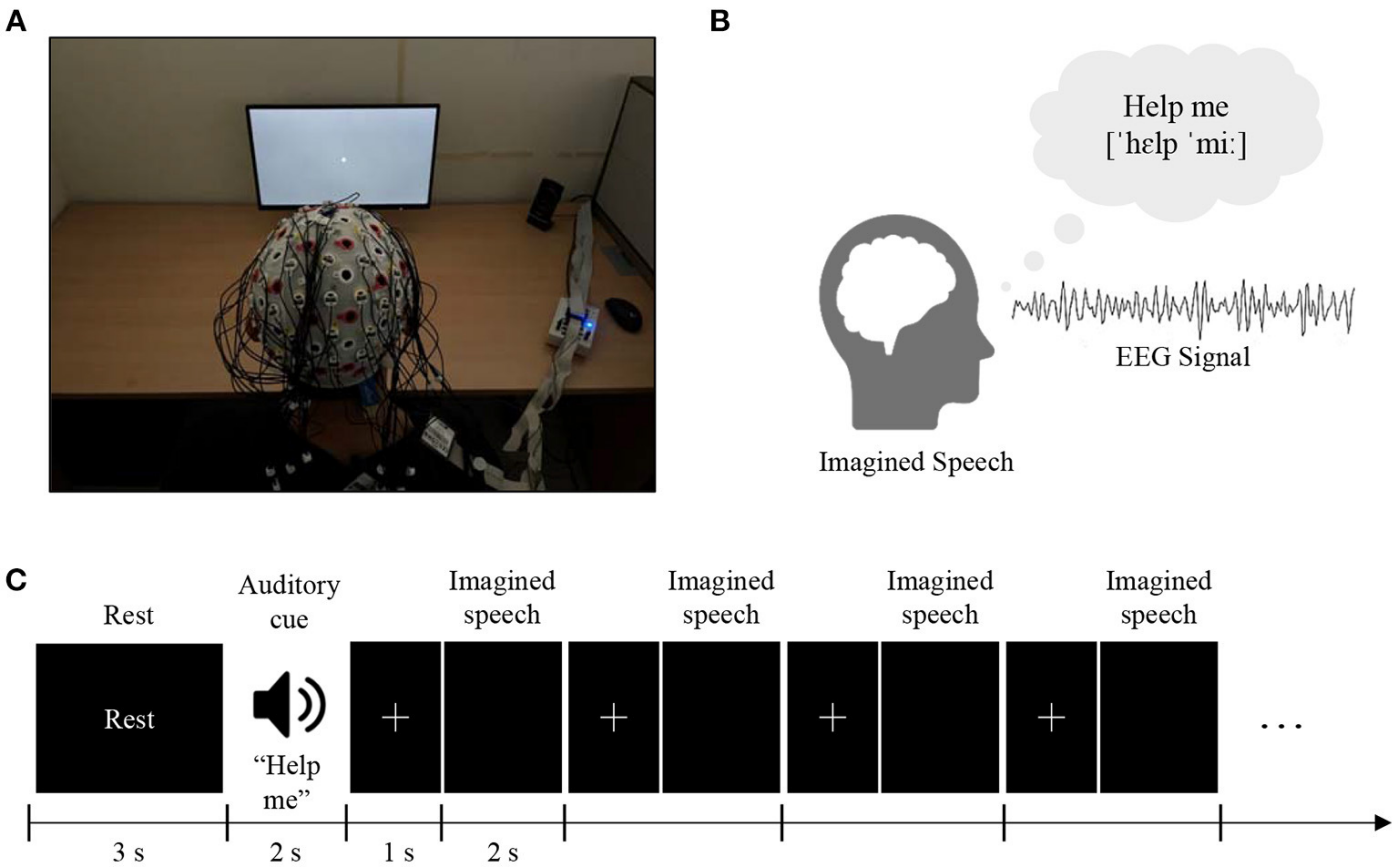
Experimental setups and protocols. **(A)** The subjects were seated in a comfortable chair in front of a 24-inch LCD monitor screen and were instructed to imagine the silent pronunciation of the given word as if they were performing real speech, without moving any articulators or making the sound. **(B)** Five critical main words/phrases for basic communication (“hello,” “help me,” “stop,” “thank you,” and “yes”) were selected. Seventy trials per class (70× 5 = 350 trials) are released for training (60 trials per class) and validation (10 trials per class) purposes. **(C)** An auditory cue was randomly presented for 2 s, followed by 0.8–1.2 s of a cross mark. The subjects were instructed to perform imagined speech of the given cue as soon as the cross mark disappeared on the screen. Four cross marks and imagined speech phases were followed in a row per random cue. After performing the imagined speech four times, 3 s of the relaxation phase was given to clear the mind.

Data Set-C is a set of EEG data related to the imagination of a person's voice conversation. The composition of this dataset represents what subjects would speak, but only in their imagination (without speaking aloud). It includes various words imagined by subjects, and the EEG signals expressed in the process were recorded. Thus, the contestants analyzed the EEG signals of the presented test dataset with the goal of classification to infer which corresponding EEG signal pattern was associated with a designated word or phrase (Figure 7).

The experimental protocol followed that of previous studies (Lee et al., 2019a, 2020a). The purpose of this experiment was to classify multi-class imagined speech with robust performance. Imagined speech of five words/phrases for basic communication (“hello,” “help me,” “stop,” “thank you,” and “yes”) was recorded from 15 subjects (S1–S15; aged 20–30 years). During the experiment, subjects were seated in a comfortable chair in front of a 24-inch LCD monitor screen. The subjects were instructed to imagine the silent pronunciation of the given word or phrase as if they were performing real speech without moving any articulators or making a sound. The subjects were directed not to perform any brain activity other than the given task. They were asked not to move and to avoid blinking while imagining or receiving the cue. All imagination trials were performed using a black screen so that subjects did not receive any stimulus in order to avoid any other factors affecting brain activity. An auditory cue representing one of the five words/phrases was randomly presented for 2 s, followed by a 0.8–1.2 s presentation of a cross mark. The subjects were instructed to perform imagined speech of the given cue as soon as the cross mark disappeared from the screen. Four cross mark (0.8–1.2 s) and imagined speech (2 s) phases were sequentially performed per random cue. After the four phases of imagined speech, a 3 s relaxation phase was allowed to clear the subject's mind for the next word/phrase.

EEG data were recorded using an signal amplifier (BrainAmp, BrainProducts GmbH, Germany). Raw data were recorded using BrainVision (BrainProducts GmbH, Germany) with MATLAB 2019a (The MathWorks Inc., USA) and 64 EEG electrodes following a 10–20 international configuration were used for the recording. The ground and reference channels were placed on Fpz and FCz, respectively. The impedance of all electrodes between the sensors and the skin of the scalp was maintained below 15 kΩ. The data disclosed to the participants for the competition were prepared by ourselves and the experimental setup was reviewed by the Korea University IRB.

#### 2.3.4. Data configuration

EEGs of five-class imagined speech words/phrases were recorded. In the experiment a total of 70 trials per class (70×5 = 350 trials) were recorded and 60 trials per class were used for training and 10 trials per class for validation purposes. Using the given validation set was not obligated. Validation of the training data could be performed not only by the given validation set, but competitors could choose a different validation method (example: N-fold cross-validation using all data). The test data were released as 10 trials per class. Detailed information regarding the released data is provided in Supplementary Table 6.

#### 2.3.5. Competition outcomes

**Figure 8:**
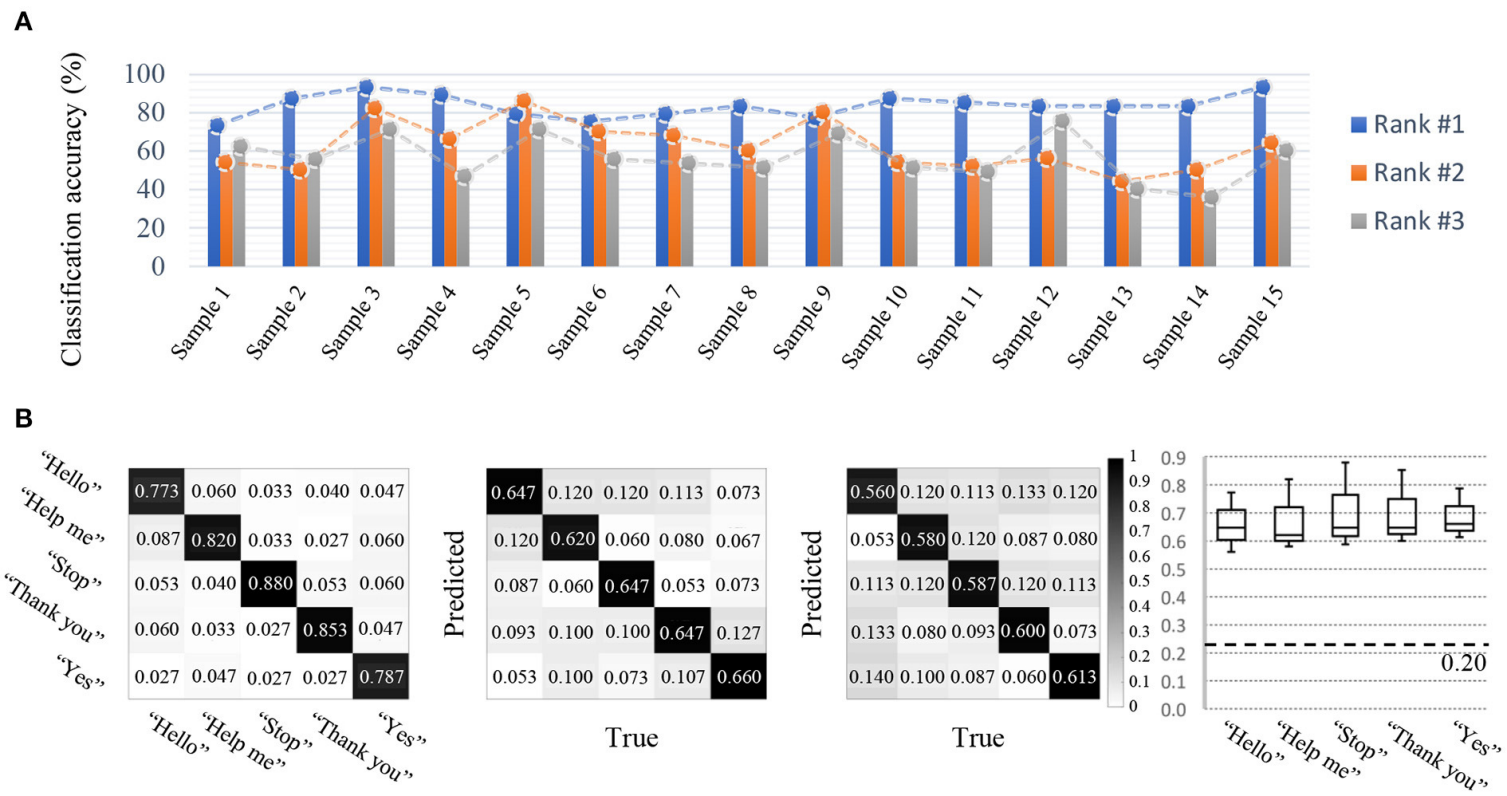
Competition outcomes. **(A)** Imagined speech BCI results (accuracy). **(B)** For imagined speech BCI, the true positive rate of every class demonstrated a true positive rate above the baseline, however, high variation was found among the different classes. In addition, the results showed different tendencies according to each participant's model.

The classification accuracy was relatively high compared to that of other tracks. In particular, a classification accuracy of 82.6% represented the best performance (Figure 8A). This indicates the possibility of imagined speech BCIs being directly used in real life in specific environments where greater performance stability may be achieved through additional models and experiments. The second-highest performance achieved 75.5% accuracy, and the difference in performance among other competitors was relatively small. Therefore, the efficiency and performance of the model created by the first-place competitor was extremely high, and the remaining competitors developed models exhibiting similar performances among their models. This indicates that the potential use of the data set provided is very high with respect to imagined speech. On the other hand, regarding the confusion matrices, true positive rates for a particular class were high, but variation was also high. In addition, the results showed different tendencies regarding true positive rates according to each participant's model (Figure 8B).

#### 2.3.6. Contribution

The competition results of imagined speech classification display a significant development in the field of intuitive BCI. Previous literature reported relatively inferior classification performance when decoding imagined speech compared to conventional and popular BCI paradigms (such as MI or P300). However, the results of the competition imply the high potential of imagined speech as another robust paradigm for BCI. Because imagined speech is a powerful paradigm, especially for conveying a user's conversational intention, the possibility of robust decoding may represent a significant milestone toward more innovative future studies on imagined speech. Further investigation of the intrinsic features of imagined speech may also contribute to improving the decoding performance along with the development of deep learning techniques using a small amount of data.

### 2.4. Data Set-D

#### 2.4.1. Background

Implementing BCI systems in real-life scenarios for people who truly need BCIs is a critical issue. BCIs require training to create a robust, accurate model so that users can use the system, and the model should be based on data collected from the same session, that is, from the same person. Moreover, consistent conditions and environments are required. Therefore, a session-dependent large dataset is required for EEG learning to produce practical BCIs. Thus, the latest methodologies have used various approaches to address these problems. Cross-session BCI research has focused on applying different approaches to solve the cross-session problem. Yang et al. implemented a discriminative feature learning strategy based on GAN for subject-independent MI data, achieving a classification accuracy of 87.8% for binary classification (Yang et al., 2021), and Kostas et al. implemented a model by applying a neural data representation based on a transformer and cross subject-oriented deep neural network model (Kostas and Rudzicz, 2020; Cao et al., 2021; Kostas et al., 2021) as described in Supplementary Table 7.

#### 2.4.2. Challenge

As mentioned above, better BCI training environments should be designed because current training environments impose a high cognitive workload on subjects owing to the long calibration times required. The goal of Data Set-D focuses on the cross-session classification of upper-limb movements. The development of session-independent BCIs aims to achieve a more accurate and robust performance even when using a limited calibration dataset for learning the decoding model. The intent is to accommodate users who must utilize a BCI system for a long time, perhaps a lifetime. Therefore, the BCI system requires technology to independently leverage such data when learning a model based on user data collected over a long period of time. In the BCI field, one of the crucial issues is solving the cross-session problem.

Decoding cross-session of upper-limb movements data is a significant challenge due to the complex dynamics of EEG signals. In particular, this classification approach is necessary for practical BCI development. The purpose of dataset D is to classify data from different sessions using training data from previous sessions based on session-independent learning. Such decoding methodology aims to train well generalizing classifiers using session-independent training data, contrary to the common practice of using session-dependent data. The expected baseline for this part was to achieve high classification performance using only data from specific subjects recorded in the past to develop practical BCI systems. For example, it is essential to build a highly accurate method for human use of BCIs. Still, simultaneously, the model must be mastered quickly and used without a session-dependent dataset. As a result, we support developing cross-session decoding techniques that are required as a condition for participants to implement practical BCIs by disclosing relevant data. To utilize the BCI system, users must participate in training sessions across each class over long periods of time. For this reason, some users performed well in the training sessions, whereas they often performed poorly in real-time sessions because of attention loss and fatigue (Abu-Rmileh et al., 2019). To further evaluate and hopefully overcome this limitation, we focused on cross-session classification using motor imagery tasks.

#### 2.4.3. Experimental protocols and paradigm

**Figure 9:**
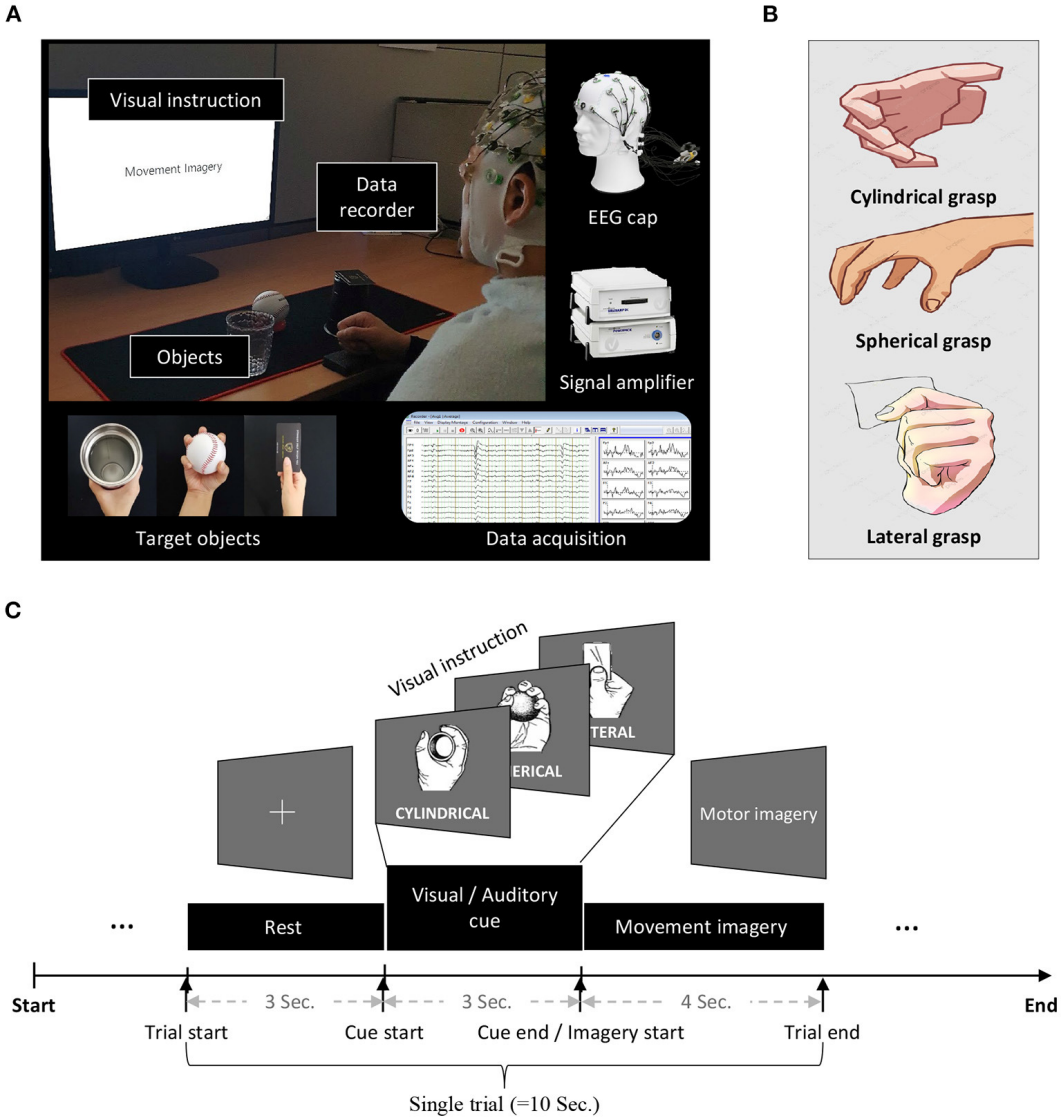
Experimental setup and protocols. Dataset was recorded under three sessions, and the datasets from the first two sessions (day 1 and day 2) was released for training purposes. The test data released later to competitors was obtained in the third session. **(A)** During a session of the experiment, subjects were seated in a comfortable chair in front of a 24-inch LCD monitor screen. **(B)** Three designated objects (cup, ball, and card) were placed on the screen, and a visual cue (a flashing green circle around the targeted object) indicated what grasping motion the subject should imagine. **(C)** A single trial comprised three continuous stages, which posed a designated task to the subjects. These experimental stages were rest, preparation, and performance of movement imagery. A single trial lasting 10 s consisted of three sub-stages, which were 3, 3, and 4 s in duration, respectively. The subject performed motor imagery during the 4 s stage after the visual cue was provided.

The subjects (S1–S15; 20–34 years of age; all right-handed) were asked to imagine three different grasping tasks: cylindrical grasp, spherical grasp, and lumbrical grasp. The dataset released at the competition to train and validate cross-session classification consisted of the following configurations based on the experimental paradigm (Figure 9). These data were collected by the authors and involved intuitive upper limb motion. The entire experiment was performed in 3-day sessions, and each session was conducted in 7-day intervals.

During a session, the subjects were asked to comfortably sit in front of a 24-inch LCD monitor (Figure 9A). Three designated objects (cup, ball, and card) were placed on the screen, and visual instructions indicated what type of grasping motion the subject should imagine. The subjects were asked to perform three intuitive imaginary grasping motions by following the visual cue (Figure 9B). The locations of the objects were randomly changed to avoid the effects of artifacts. Each subject performed 50 trials per grasping action (150 trials: 3 classes × 50 trials). We asked the subjects to imagine a specific grasp only once during the motor imagery period of 4 s.

A single trial comprised three continuous stages, which posed a designated task to each subject. These stages were rest, preparation, and performance of the motor imagery cue. A single trial lasted for 10 s and consisted of three sub-stages, which were 3, 3, and 4 s in length, respectively. The subjects performed motor imagery during the final stage for 4 s after the visual cue was provided (Figure 9C).

The EEG data were recorded using an EEG signal amplifier (BrainAmp, Brain Products GmbH, Germany), sampled at 250 Hz with a 60 Hz notch filter. Raw data were recorded using BrainVision (Brain Products GmbH, Germany) with MATLAB 2019a (The MathWorks Inc, USA). Sixty EEG electrodes were selected by following the 10–20 international configuration (Fp1-2, AF5-6, AF7-8, AFz, F1-8, Fz, FT7-8, FC1-6, T7-8, C1-6, Cz, TP7-8, CP1-6, CPz, P1-8, Pz, PO3-4, PO7-8, POz, O1-2, Oz, and Iz). The ground and reference channels were placed on Fpz and FCz, respectively. The impedance of all electrodes between the sensors and the skin of the scalp were maintained below 15 kΩ. Data disclosed to competition participants were prepared by the authors and the experiment was reviewed by the Korea University IRB.

#### 2.4.4. Data configuration

Data Set-D was recorded over three sessions, and data from the first two sessions (day 1 and day 2) were released for training and validation purposes, respectively. The test data were obtained from the third session (day 3). Because the purpose of this stage of the competition was to evaluate the performance between sessions, the size of the training and verification data was the same. That is, data from 150 trials obtained in the day 1 session were designated as training data, and data from another 150 trials obtained on day 2 were used as the verification data. Data obtained on day 3 were released later during the competition as the test data. Participants were allowed to use data obtained from the second day for validation purposes, but were also allowed to combine the data with the original training data obtained on day 1 to prepare 300 trials as the training dataset. In total, including data from day 1 and data from day 2, the dataset consisted of 150 trials for training and 150 trials for the validation set. Finally, we evaluated the cross-session classification results using data from the day 3 session (150 trials) as the test set. The description of the data is presented in Supplementary Table 8.

#### 2.4.5. Competition outcomes

**Figure 10:**
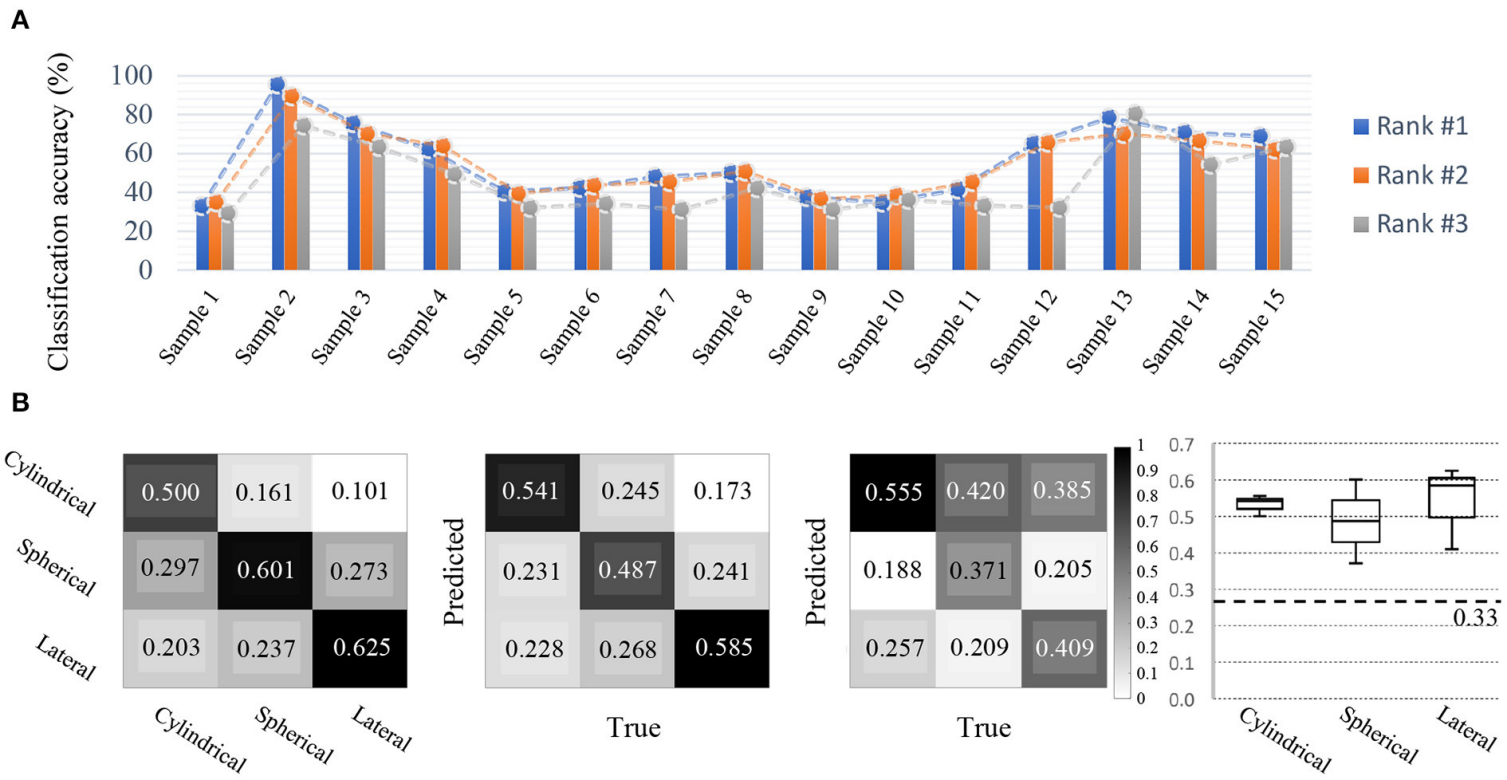
Competition outcomes. **(A)** Cross-session BCI results (Accuracy). Compared to other disciplines, all participants achieved relatively good decoding performance. For cross-session BCI, various grasping MIs using a single-arm were performed, and the top-3 BCI model performances during various sessions were acceptable per each class. **(B)** Confusion metrices for each class and candle plot for comparison. Based on the results of the top-3 participants, the prediction by class and the true answer rate are organized into confusion metrics. From the left, metrices showing which class was predicted more accurately by Rank 1, Rank 2, and Rank 3 participants. The candle plot on the far right corresponds to classes Cylindrical, Spherical, and Lateral, respectively, from the left, and representing the mean and standard deviation of the classification results achieved by the top-3 participants by class.

Consequently, contestants were given the opportunity to challenge not only data from the cross-session subjects, but also data that had previously produced good results independently of the three sessions using their proposed decoding method. All participants competed in their own independent environments, but the performances of the models created by most participants were similar, as shown in Figure 10A. The results were judged up to the top-5 competition groups who developed meaningful decoding models and their results were analyzed in depth.

The performance of the models created by the participants to develop the cross-session BCI showed the following classification results. For the top two competitors, the decoding models achieved significantly higher classification performance between classes compared to other competitors. The other participants created a model using the training set that was provided, and later used the test set to validate the models that they created, and scores were relatively similar among participants. For example, in the data involving samples from 15 subjects, the participants created models that exhibited high levels of performance for samples 2, 3, 13, and 15 (> 0.60), but all models exhibited low performance for the remaining samples (Figure 10B). However, in the case of the participant who achieved first place, classification performance was achieved well for all subjects, which indicates that they created a model that performed more reliably. In addition, true positive rates were fairly distributed for each class across all models in the top-5 rankings.

#### 2.4.6. Contribution

Robust decoding performance in cross-session problems is critical toward developing practical BCI technology because general users do not wish to have to train or calibrate a BCI system every time that they use it in real life. The main problem in creating robust models for cross-session purposes is that models learned based on data obtained over a specific period of time do not work as well on data obtained over other periods owing to uncertainties and inconsistent features in EEG data. Therefore, the competition conducted using Data Set-D deals with critical approaches to create a stable decoding model or methodology for the development of practical BCI devices that can be worn by users in their daily lives.

### 2.5. Data Set-E

#### 2.5.1. Background

Making BCI available in a mobile environment is an essential requirement for creating a practical BCI system. For BCI technology to be deeply incorporated into our daily lives, reliable performance must be achieved in situations involving movement, and the ability to use the system continuously must be secured. Noninvasive BCIs are necessarily susceptible to noise because they are based on the principle of collecting various distorted EEG signals with electrodes attached to the scalp and removing the noise to analyze the signals. During movement involving EEG recorders, noise occurs primarily because of the movement of the EEG electrodes due to poor contact, vibration, and muscle movement, which hinder performance of the BCI. However, newer BCI techniques have been developed that allow the construction of models that effectively eliminate noise in mobile environments and achieve stable and accurate decoding performance (Lee Y. E. et al., 2021). Moreover, comfortable hardware interfaces have been developed, such as an ear-EEG, which compensate for the shortcomings of scalp-EEGs (requiring annoying gels that are difficult to wash out) (Debener et al., 2015; Bleichner and Debener, 2017). Ambulatory BCI for stable performance in mobile environments has been discussed in recent studies (Lee et al., 2020c; Chuang et al., 2021). Supplementary Table 9 lists some of the systems developed.

#### 2.5.2. Challenge

Ambulatory BCI and practical BCIs outside the laboratory are essential issues in BCI technology. Decoding EEG signals under ambulatory environments are challenging due to the numerous artifacts. Moreover, decoding a small number of channel EEG signals for higher practicality is a challenge due to its low performance. Ear-EEG is a representative of simple and practical hardware that records brain signals, which has poor performance compared to typical scalp-EEG. Therefore, in this section, the issue of ambulatory and practical BCI was investigated by decoding EEG including ear-EEG during ERP tasks in the ambulatory environment. To widely spread practical BCI technology, we should consider the use of EEG in the real world. Several state-of-the-art BCI systems have demonstrated increased system performance using deep learning (Lee and Lee, 2020; Lee et al., 2020d; Mammone et al., 2020; Sun et al., 2020), but generally evaluate the system only in laboratory environments. However, some technical problems with external and internal artifacts have been addressed in real-world environments. That is, even if a BCI system with stable performance is developed, a system that can be used only in a laboratory environment is not suitable for real-world use. Therefore, the problem of improving decoding performance through artifact removal in an ambulatory environment has always been a challenge for advancing BCIs and has not yet been completely solved (Lee et al., 2020c). Thus, for practical BCIs, it is necessary to develop artifact removal techniques in the preprocessing stage and create robust decoding models while using simple hardware such as an ear-EEG system. Hence, in the competition, one of the goals was for participants to propose novel approaches for an ambulatory environment. More specifically, enhancing the performance of the event-related potential (ERP) classification using scalp-EEG and ear-EEG during walking at a 1.6 m/s pace.

#### 2.5.3. Experimental protocols and paradigm

**Figure 11:**
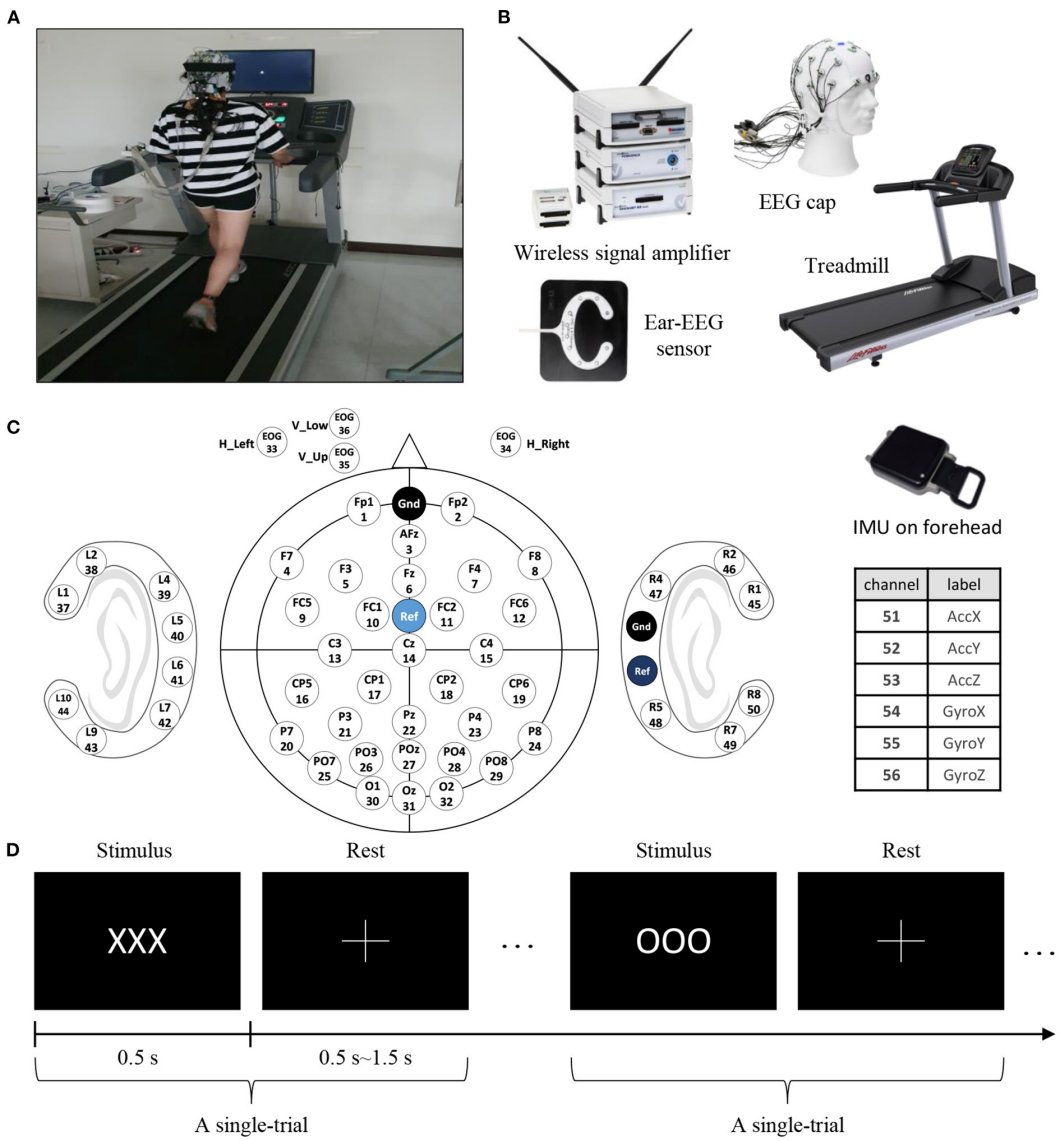
Experimental setups and protocols. **(A)** Experimental setup showing a subject walking on a treadmill. **(B)** In this experiment, we simultaneously collected data from various devices: EEG signals from the scalp (actiCap, BrainProducts GmbH, Germany), EEG signals from around the ear (cEEGrid, TMSi, USA), forehead IMU signals (APDM, APDM wearable technologies, USA), and from the treadmill. **(C)** Channel labels: 32 scalp-EEG electrodes, 3 EOG electrodes, and 6 IMU sensors. **(D)** The experimental paradigm was executed with target (“OOO”) and non-target (“XXX”) characters. The ratio of targets was 0.2 and the number of total trials was 300. In a trial, a stimulus was presented for 0.5 s followed by the cross symbol indicating a random rest period lasting 0.5–1.5 s.

This dataset consists of scalp-EEG, ear-EEG, and inertial measurement unit (IMU) data from 15 subjects. The subjects (S1–S15, aged 19–32 years, 11 males and 4 females) were asked to perform ERP paradigm and walk in 1.6 m/s (Figure 11A). We recorded scalp-EEG, ear-EEG, EOG, and IMU sensor data. The data using these devices were collected as follows: scalp-EEG electrodes (channel numbers: 1–32), EOG electrodes (channel numbers: 33–36), ear-EEG electrodes (channel numbers: 37–50), and IMU sensor (channel numbers: 51–56) (Figures 11B,C).

In the controlled environment, the subjects could walk and move on a treadmill, and we collected EEG signals that occur in moving environments from a sufficient number of subjects (Figure 11A). The participants repeatedly stood and walked at 1.6 m/s when using the treadmill placed in front of a 24-inch LCD monitor screen.

ERP is an electrical potential induced in the central and parietal cortices in response to particular cognitive tasks. Attention to the target induces P300 components that have task-relevant peaks that occur 300 ms after a target stimulus. In this experiment, this paradigm was executed using target (‘OOO') and non-target (‘XXX') characters. The target ratio was 0.2, and the total number of trials was 300. In a trial, the stimulus was presented for 0.5 s and a cross was shown to indicate a rest period randomly lasting 0.5–1.5 s (Figure 11D). The data provided to the participants for the competition were prepared by the authors.

#### 2.5.4. Data configuration

Each trial was segmented from –200 to 800 ms, depending on the stimulus presentation time. The data from 300 trials were divided into training, validation, and test sets using 0.6, 0.2, and 0.2 ratios, respectively. Of the 300 trials, we divided the first 180 trials as training, the next 60 trials as validation, and the final 60 trials as test sets. Only the training and validation sets were provided to competitors in order to obtain a fair evaluation. The data configurations for both the scalp-EEG and ear-EEG were obtained under the same conditions. The data are presented in Supplementary Table 10.

#### 2.5.5. Competition outcomes

**Figure 12:**
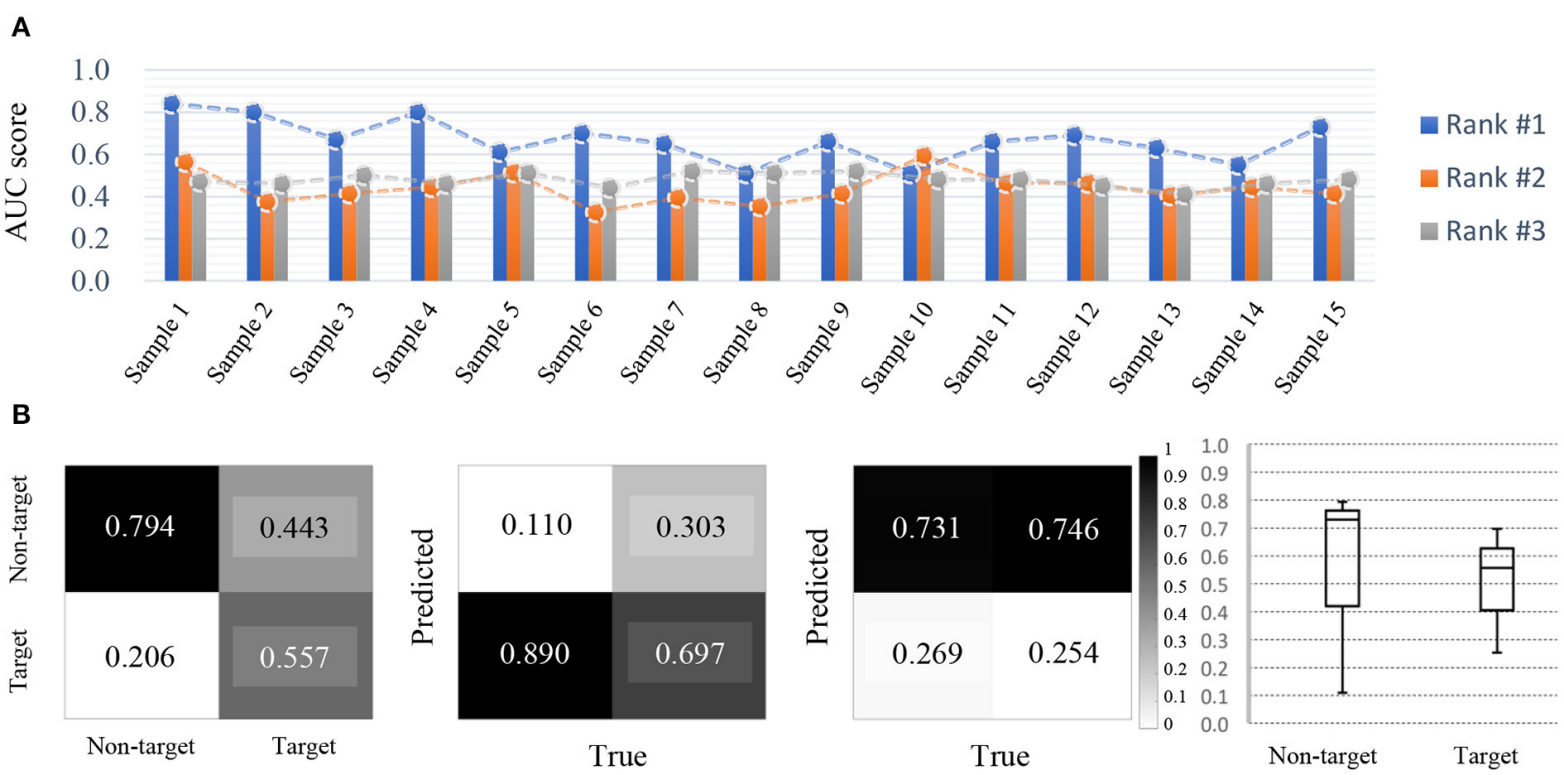
Competition outcomes. **(A)** Ambulatory BCI results (AUC score). Rank #1 and #2 show relatively large performance deviation for each sample. **(B)** Overall, the results tended to distinguish between target and non-target class, and rank #1 showed high AUC scores in the ambulatory environment.

The competitors performed the task of decoding ERP signals in an ambulatory environment at a speed of 1.6 m/s and detection performances were evaluated based on area under the curve (AUC) using a reliable noise abatement method. The highest AUC was 0.728, and the lowest was 0.506, in the top-3 ranks. The performances among the models created by the participants showed large variations (Figure 12A). In addition, the two top models exhibited large differences among subject samples (Figure 12A). However, the top model achieved a high true positive rate between the target and non-target classes, which were classified on an imbalanced dataset (Figure 12B). Compared to other tracks, there was no significant difference among the performances of the models developed by the participants. The top-scoring model achieved the highest true positive rates of 0.794 and 0.557 for each class, but the differences in average performance were not significant among participants with the best performances.

#### 2.5.6. Contribution

Although EEG is the simplest way to record brain activity, EEG data are vulnerable to noise and devices are annoying and inconvenient to wear. Ambulatory BCI research is essential for developing practical BCI systems based on artifact removal methods, robust decoding models, and a simple hardware interface. The BCI competition involving Data Set-E can provide a foothold for the development of useful practical BCI technologies. Through this competition, the importance of the mobile environment BCI technology was further promoted, and the technological performance of the participants was significantly increased owing to the significant developments regarding this technology. The decoding technology based on a simple hardware interface has improved in performance to the point that it is not inferior to conventional scalp-EEG decoding technology, which demonstrates the possibility of real-life applications. Therefore, we expect the development of a system using EEG that achieves high performance in real-world ambulatory applications.

## 3. Discussion

In this review, we evaluated possible solutions to these five aforementioned issues, including few-shot EEG learning for short-calibration, Micro-sleep detection from a single channel, Imagined speech decoding for intuitive BCI communication, Cross-session classification of upper-limb movements, and EEG(+ear-EEG)-based ERP detection under ambulatory environment, throughout the 2020 International BCI competition conducted jointly with the 9^*th*^ International Winter Conference on Brain–Computer Interface 2021. Through the competition, we have confirmed that the presented issues were appropriate for assessing advances in BCIs; nevertheless, several technical concerns remain. For example, many scholars have solved few-shot learning, domain generalization, and cross-session problems with high levels of performance in other disciplines (Seo et al., 2020; Zhou K. et al., 2020; Kim G. et al., 2021; Kwon and Im, 2021; Li et al., 2021). BCI systems using intuitive speech imagination, also compared to those that require the imagination of existing behavior or visual external stimuli, have been found to lack sufficient decoding performance (Cooney et al., 2018; Lee et al., 2020a).

To adapt BCI technology to real-world applications, many investigators have applied advanced machine learning to improve BCI performance and solve chronic problems (e.g., the number of classes, real-time implementation, and invariance between subjects) (Kwon et al., 2019; Vidaurre et al., 2021; Zhang et al., 2021). In fact, to solve the problems presented in the competition, only one group implemented a revised machine learning technique, whereas other participants presented their own deep learning architectures. Recent research demonstrating improved classification accuracy using deep learning methods has been reported. The most recent work applying neural networks has focused on the development of various architectures and algorithms while testing them on a standard benchmark (Craik et al., 2019; Stieger et al., 2021) or datasets gathered by individual researchers. Some researchers have recently begun to validate these methods on their own datasets in online scenarios (Tabar and Halici, 2016; Jeong et al., 2020c). However, compared to traditional machine-learning methods, deep learning-based approaches still have several problems when applied to BCI. Among the concerns are lack of high-quality data and large amount of data requirements, long training time, and developing methods that can be used to improve performance (Jeong et al., 2020c). Therefore, the use of BCI technology in daily life based on machine/deep learning models that achieve high performance should consider designing simple, compressed models. Furthermore, these more advanced models and architectures need to be explainable (Sturm et al., 2016) and tested for artifacts and Clever Hans effects (Lapuschkin et al., 2019; Anders et al., 2022). Through this review, we hope that many investigators will be motivated to focus on these aspects in the future and that they utilize both the advantages of machine learning and deep learning to contribute to further BCI advances.

In addition, it is essential to ensure the portability of hardware to conveniently use applications equipped with BCI technology in real-world environments. Difficulties involve not only portability issues but the types of signal sensing devices (e.g., wet, dry, and semi-dry) and difficulties associated with wearing sensor caps (Popescu et al., 2007; von Lühmann et al., 2016; Schwarz et al., 2020a; Kim J-Y. et al., 2021). Therefore, BCI investigators will have to consider not only software but hardware aspects for advancing BCI in the future.

This review also aims to show some of the limitations of the current technology and provide open problems for researchers in the field. Furthermore, the competition was viewed as an opportunity to provide a standardized assessment of the progress of current BCI technologies.

## 4. Conclusion

In conclusion, this review presents some of the prominent BCI challenges faced by investigators. We evaluated the technical level of the models applied to highly complex problems that BCIs face through the 2020 International BCI competition. Based on these results, it is apparent that further developments are necessary for broader use of BCI and its commercialization. A sustained advancement in BCI technology will furthermore help to obtain insights into fields such as cognitive neuroscience and clinical diagnostics. Thus, we hope that BCI investigators will be inspired to explore future development directions and advance current levels of BCI technology through the released datasets. BCI technology, which is still in a relatively adolescent phase compared to other scientific fields, requires significant exploration for further development. Through competitions, we believe that convergence between various interdisciplinary fields will occur naturally and will thus contribute to the exchange of new techniques and ideas that will benefit different scientific fields.

## Author contributions

J-HJ, Y-EL, S-HL, G-HS, Y-SK, and J-HC prepared the competition dataset and conducted the meta-analysis. JM, K-RM, and S-WL guided the review of the conceptualized topics. All authors contributed to a discussion of the contents, reviewed, and edited the manuscript.

## Funding

This research was partly supported by an Institute of Information & Communications Technology Planning & Evaluation (IITP) grant, funded by the Korea government (No. 2017-0-00451) and also this work was supported by the research grant of the Chungbuk National University in 2022.

## Conflict of interest

The authors declare that the research was conducted in the absence of any commercial or financial relationships that could be construed as a potential conflict of interest.

## Publisher's note

All claims expressed in this article are solely those of the authors and do not necessarily represent those of their affiliated organizations, or those of the publisher, the editors and the reviewers. Any product that may be evaluated in this article, or claim that may be made by its manufacturer, is not guaranteed or endorsed by the publisher.
