# Supplementary material for: 2020 International brain–computer interface competition: A review
Source: Front Hum Neurosci. 2022 Jul 22;16:898300. doi: 10.3389/fnhum.2022.898300 (PMC9354666; doi:10.3389/fnhum.2022.898300)
Supplement: Supplementary file 1 [file Data_Sheet_1.PDF]

Supplementary Material

1 SUPPLEMENTARY TABLES

Table S1. Summary of state-of-the-art studies related to Data Set-A

References	Objective	Dataset	Methods	Performance measurement
(Azab et al., 2019)	Propose a transfer learning from MI-EEG	Local<br>BCI competition IV 2a<br>BCI competition III IVa	S-wLTL<br>Us-wLTL	71.0% (Acc.): Local<br>75.6% (Acc.): BCI competition IV 2a<br>75.0% (Acc.): BCI competition III Iva
(Singh et al., 2019a)	Reduce calibration time for MI	BCI Competition III	SPD matrics	86.1% (Acc.)<br>0.72 (Kappa value)
(Singh et al., 2019b)	Classify small sample MI data	BCI Competition III	Regularized MDRM	87.2% (Acc.)<br>0.74 (Kappa value)
(McCartney et al., 2019)	Reduce short-calibration time for BCI	Trento dataset<br>Stanford dataset	Visuo-semenatic<br>feature representation	61.3% (Acc.)<br>62.2% (Acc.)
(Zhang et al., 2020)	Adapt the calibration with fewer training data	BCI competition IV	HDNN-TL	0.80 (Kappa value)
(An et al., 2020)	Classify MI based on few-shot learning	BCI competition IV 2b	Relation network	74.6% (Acc.)
(Shim et al., 2020)	Decode intuitive MI based on few-shot learning	Local	Gradual relation network	80.8% (Acc.)

Abbreviations: Acc., accuracy; S-wLTL, supervised weighted logistic regression-based transfer learning; Us-wLTL, unsupervised weighted logistic regression-based transfer learning; SPD, symmetric positive definite; MDRM, minimum distance to Riemannian mean; HDNN-TL, hybrid deep neural network with transfer learning

Table S2. Description of the Data Set-A (training, validation, and test), including event types, channel labels, and configuration of data format.

**Sets**	**Training & Validation & Test**							
**Class labels**	Event				Class			
	1				Right-hand			
	2				Left-hand			
**Channel labels**	No.	Name	No.	Name	No.	Name	No.	Name
	1	Fp1	21	CP6	41	CP4	61	PO3
	2	Fp2	22	TP10	42	P1	62	PO4
	3	F7	23	P7	43	P2		
	4	F3	24	P3	44	POz		
	5	Fz	25	Pz	45	FT9		
	6	F4	26	P4	46	FTT9h		
	7	F8	27	P8	47	TTP7h		
	8	FC5	28	PO9	48	TP7		
	9	FC1	29	O1	49	TPP9h		
	10	FC2	30	Oz	50	FT10		
	11	FC6	31	O2	51	FTT10h		
	12	T7	32	PO10	52	TPP8h		
	13	C3	33	FC3	53	TP8		
	14	Cz	34	FC4	54	TPP10h		
	15	C4	35	C5	55	F9		
	16	T8	36	C1	56	F10		
	17	TP9	37	C2	57	AF7		
	18	CP5	38	C6	58	AF3		
	19	CP1	39	CP3	59	AF4		
	20	CP2	40	CPz	60	AF8		
**Configuration**	Name				Description			
	epo.x				Raw data			
	epo.y				Class labels			
	epo.fs				Sampling frequency			
	epo.t				Time points			
	epo.className				Class name			
	epo.file				File name			
	epo.clab				Channel information			
	mnt.x				X coordinates for channel position			
	mnt.y				Y coordinates for channel position			
	mnt.pos_3d				3D coordinates for channel position			
	mnt.clab				Channel information			

Table S3. Summary of state-of-the-art studies related to Data Set-B

References	Objective	Dataset	Methods	Performance measurement
(Shahin et al., 2017)	Sleep stage classification (insomnia-impacted)	University Hospital of Berlin	DNN	92.0% (Acc.)
(Chambon et al., 2018)	Sleep stage classification	MASS	DNN	0.81 (Acc.)
(Mousavi et al., 2019)	Sleep stage classification	Sleep-EDF	SleepEEGNet	84.2% (Acc.)<br>0.79 (Kappa value)
(Perslev et al., 2019)	Sleep stage classification	ISRUC<br>Sleep-EDF	U-Time	0.77 (F1 score): ISRUC<br>0.76 (F1 score): Sleep-EDF
(Yan et al., 2020)	Sleep stage classification	SHHS<br>ISRUC<br>Sleep-EDF	DNN	0.87 (Acc), 0.81 (Kappa value): SHHS<br>0.86 (Acc), 0.82 (Kappa value): ISRUC<br>0.86 (Acc), 0.81 (Kappa value): Sleep-EDF
(Gao et al., 2019)	Driver fatigue estimation	Local	ESTCNN	97.4% (Acc.)
(Wu et al., 2020b)	Real-time fatigue detection using EEG	Local	Treelet-WiGMM	92.6% (Acc.)
(Karuppusamy and Kang, 2020)	Drvier fatigue detection using multi-modality	Local	Multimodal DNN	93.9% (Acc.)
(Lin et al., 2020)	Drvier fatigue detection	Local	4-D CNN	0.56 (RMSE)<br>0.27 (CC)
(Wu et al., 2020a)	Cognitive status detection	Local	GDBN	93.3% (Acc.)
(Ko et al., 2021)	Drowsiness classification using EEG	SEED-VIG	MSNN	5.38 (Number of false positive)

Abbreviations: Acc., accuracy; PSD, power spectral density; DNN, deep neural network; SHHS, sleep heart health study; ESTCNN, EEG-based spatial–temporal convolutional neural network; RMSE, root mean square error; Treelet-WiGMM, Treelet-warped infinite Gaussian mixture model; GDBN, gamma deep belief network; MSNN, Multi-scale neural network

Table S4. Description of the Data Set-B (training, validation, and test), including event types, channel labels, and configuration of data format.

Sets	Training		Validation & Test	
**Class labels**	Event	Class	Event	Class
	0	Wakefulness (W)	0	Wakefulness (W)
	1	NREM1 (1)	1	Microsleep (M)
	2	NREM2 (2)		
	3	NREM3 (3)		
	4	NREM4 (4)		
	5	REM (R)		
	6	Movement (M)		
**Channel label**	No.	Name	No.	Name
	1	Pz-Oz	1	Pz-Oz
**Configuration**	Name	Description	Name	Description
	epo.x	Raw data	epo.x	Raw data
	epo.fs	Sampling frequency	epo.fs	Sampling frequency
	epo.y	Class labels	epo.y	Class labels
	mnt.clab	Channel information	mnt.clab	Channel information

Table S5. Summary of state-of-the-art studies related to Data Set-C

References	Objective	Dataset	Methods	Performance measurement
(Nguyen et al., 2017)	Imagined speech decoding from EEG	Local	CSP+RVM	48.9% (Acc.)
(Sousa et al., 2017)	Controlling BCI systems using pure VI	Local	SVM	87.6% (Acc.)
(Moctezuma et al., 2019)	Exploring a new biometric measure using imagined speech	Local	IWE+RF	95.0% (Acc.)
(García-Salinas et al., 2019)	Transfer learning for imagined speech EEG	“Torres-García" dataset	CSP+NBC	65.5% (Acc.)
(Tian et al., 2018)	Discovering the imagined speech features	Local	One-way ANOVA	<0.001 (*p*-value)
(Saha and Fels, 2019)	Imagined speech decoding from EEG	“Nyuyen" dataset	Mixed DNN	84.2% (Acc.)
(Lee et al., 2020a)	Imagined speech and visual imagery decoding from EEG	Local	CSP+SVM	39.7% (Acc.): Imagined speech<br>40.1% (Acc.): Visual imagery
(Kristensen et al., 2020)	Comparing VIm and VInt from EEG	Local	CSP+SVM	88.9% (Acc.): VIm<br>91.1% (Acc.): VInt
(Cooney et al., 2020)	Imagined speech decoding from EEG	LIRINS dataset	CNN	40.9% (Acc.): Imagined words<br>44.4% (Acc.): Imagined vowels
(Kaongoen et al., 2021)	Imagined speech decoding using ear-EEG	Local	MLELM	38.2% (Acc.)
(Li et al., 2021b)	Imagined speech decoding from EEG	Local	HS-STDCN	54.3% (Acc.)

Abbreviations: Acc., accuracy; CSP, common spatial pattern; RVM, relevance vector machine; VI, visual imagery; SVM, support vector machine; IWE, instantaneous wavelet energy; RF, random forest; NBC, naive Bayes classifier; ANOVA, analysis of variance; DNN, deep neural network; VIm, vocal imagery; VInt, vocal intention; CNN, convolutional neural network; MLELM, multi-layer extreme learning machine; HS-STDCN, hybrid-scale spatial-temporal dilated convolution network

Table S6. Description of the Data Set-C (training, validation, and test), including event types, channel labels, and configuration of data format.

Sets	Training & Validation & Test							
**Class labels**	Event				Class			
	1				Hello			
	2				Help me			
	3				Stop			
	4				Thank you			
	5				Yes			
**Channel labels**	No.	Name	No.	Name	No.	Name	No.	Name
	1	Fp1	17	TP9	33	AF7	49	C2
	2	Fp2	18	CP5	34	AF3	50	C6
	3	F7	19	CP1	35	AF4	51	TP7
	4	F3	20	CP2	36	AF8	52	CP3
	5	Fz	21	CP6	37	F5	53	CPz
	6	F4	22	TP10	38	F1	54	CP4
	7	F8	23	P7	39	F2	55	TP8
	8	FC5	24	P3	40	F6	56	P5
	9	FC1	25	Pz	41	FT9	57	P1
	10	FC2	26	P4	42	FT7	58	P2
	11	FC6	27	P8	43	FC3	59	P6
	12	T7	28	PO9	44	FC4	60	PO7
	13	C3	29	O1	45	FT8	61	PO3
	14	Cz	30	Oz	46	FT10	62	POz
	15	C4	31	O2	47	C5	63	PO4
	16	T8	32	PO10	48	C1	64	PO8
**Configuration**	Name				Description			
	epo.x				Raw data			
	epo.y				Class labels			
	epo.fs				Sampling frequency			
	epo.t				Time points			
	epo.className				Class name			
	epo.file				File name			
	epo.clab				Channel information			
	mnt.x				X coordinates for channel position			
	mnt.y				Y coordinates for channel position			
	mnt.pos_3d				3D coordinates for channel position			
	mnt.clab				Channel information			

Table S7. Summary of state-of-the-art studies related to Data Set-D

References	Objective	Dataset	Methods	Performance measurement
(Zhang et al., 2019)	Movement intention detection from session-to-session EEG data	Local	G-HAM	76.3% (Acc.)
(Fahimi et al., 2020)	Improve cross-session BCI performance	Local	DCGAN	85.5% (Acc.)
(Luo et al., 2020)	Enhance cross-session BCI performance	SEED dataset<br>DEAP dataset	sWGAN	92.2% (Acc.): SEED dataset<br>50.4% (Acc.): DEAP dataset
(Kostas and Rudzicz, 2020)	Decoding cross-session EEG data to improve versatility	BCI competition dataset<br>MMI dataset<br>P300 dataset<br>ERN dataset	TIDNet	77.3% (Acc.): BCI competition dataset<br>42.6% (Acc.): MMI dataset<br>0.854 (AUROC): P300 dataset<br>0.794 (AUROC): ERN dataset
(Freer and Yang, 2020)	Decode self-paced motor imagery from cross-session EEG data	BCI competition dataset	C-LSTM	0.654 (F1-score)
(Yang et al., 2021)	Decode session-to-session MI data	BCI competition dataset	GAN	87.8% (Acc.)
(Kostas et al., 2021)	Learn massive EEG data for session independent BCI	Pre-training: TUEG dataset<br>Downstream: MMI, BCIC, ERN, P300, SSC	BENDR	86.7 (BAC): MMI dataset<br>42.6% (Acc.): BCIC dataset<br>0.65 (AUROC): ERN dataset<br>0.72 (BAC): SSC dataset<br>0.72 (AUROC): P300 dataset
(Zhang et al., 2021)	Transfer DCNN model for motor imagery classification	OpenBMI dataset	Adaptive transfer learning	84.1% (Acc.): Subject-independent<br>86.8% (Acc.): Subject-adaptive
(Li et al., 2021a)	Decode cross-session motor imagery	BCI competition dataset	CMS+EEGNet	75.6% (Acc.)

Abbreviations: Acc., accuracy; G-HAM, graph-based hierarchical attention model; DCGAN, Deep convolutional generative adversarial network; sWGAN, selective Wassestein generative adversarial network; TIDNet, thinker-invariant denseNet-inspired DNN; C-LSTM, convolutional long-short term memory; GAN, generative adversarial network; BENDR, BERT-inspired neural data representation;BAC, class balanced accuracy; AUROC, area under the receiver operating characteristic; CMS, component-mixing strategy

Table S8. Description of the Data Set-D (training, validation, and test), including event types, channel labels, and configuration of data format.

**Sets**	**Training & Validation & Test**							
**Class labels**	Event				Class			
	1				Cylindrical			
	2				Spherical			
	3				Lateral			
**Channel labels**	No.	Name	No.	Name	No.	Name	No.	Name
	1	Fp1	16	C3	31	POz	46	T8
	2	AF7	17	C1	32	Fp2	47	CP2
	3	AF3	18	Cz	33	AF4	48	CP4
	4	AFz	19	TP7	34	AF8	49	CP6
	5	F7	20	CP5	35	F2	50	TP5
	6	F5	21	CP3	36	F4	51	P2
	7	F3	22	CP1	37	F6	52	P4
	8	F1	23	CPz	38	F8	53	P6
	9	Fz	24	P7	39	FC2	54	P8
	10	FC7	25	P5	40	FC4	55	PO4
	11	FC5	26	P3	41	FC6	56	PO8
	12	FC3	27	P1	42	FT8	57	O1
	13	FC1	28	Pz	43	C2	58	Oz
	14	T7	29	PO7	44	C4	59	O2
	15	C5	30	PO3	45	C6	60	Iz
**Configuration**	Name				Description			
	epo.x				Raw data			
	epo.y				Class labels			
	epo.fs				Sampling frequency			
	epo.file				File name			
	epo.clab				Channel information			
	mnt.x				X coordinates for channel position			
	mnt.y				Y coordinates for channel position			
	mnt.pos_3d				3D coordinates for channel position			
	mnt.clab				Channel information			

Table S9. Summary of state-of-the-art studies related to Data Set-E

References	Objective	Dataset	Methods	Performance measurement
(Arad et al., 2018)	Removal motion artifacts during locomotion	Local	AMICA	<0.001 (*p*-value)
(Nordin et al., 2019b)	Removal motion artifacts while over the obstacle	Local	Dual-layer EEG validation	0.92 (r-value)
(Nordin et al., 2019a)	Determine walking speed affects in EEG	Local	PCA+CCA	<0.05 (p-value)
(Malcolm et al., 2019)	Evaluate test-retest ERP while walking	Local	Mean amplitude	>0.63 (ICC)
(Kilicarslan and Vidal, 2019)	Removal motion artifacts from EEG	Local	Adaptive de-noising framework	<0.40 (CC)
(Lee et al., 2020b)	Decode ERP/SSVEP on a treadmil	Local	cIOL	0.82 (AUC): 0.8m/s speed<br>0.73 (AUC): 1.6m/s speed<br>0.64 (AUC): 2.0m/s speed
(Sun et al., 2020)	Removing artifacts of seizure data	CHB-MIT database	1D-ResCNN	0.0819 (RMSE): ECG 0dB<br>23.96 (SNR): ECG 0dB
(Chuang et al., 2021)	Removing artifacts of resting state in real-world	Local	IC-U-Net	0.57 (MSE): eye<br>4.06 (SNR): eye

Abbreviations: Acc., accuracy; AMICA, adaptive mixture independent component analysis; PCA, principal component analysis; CCA, canonical correlation analysis; ICC, intraclass correlation coefficient; IOL, constrained independent component analysis with online learning; AUC, area under curve; RMSE, root mean square error; SNR, signal-to-noise ratio

Table S10. Description of the Data Set-E (training, validation, and test), including event types, channel labels, and configuration of data format.

**Sets**	**Training & Validation & Test**							
**Class labels**	Event				Class			
	1				Non-target			
	2				target			
**Channel labels**	No.	Name	No.	Name	No.	Name	No.	Name
	1	Fp1	15	C4	29	PO8	43	L9
	2	Fp2	16	CP5	30	O1	44	L10
	3	AFz	17	CP1	31	Oz	45	R1
	4	F7	18	CP2	32	O2	46	R2
	5	F3	19	CP6	33	HEOGL	47	R4
	6	Fz	20	P7	34	HEOGR	48	R5
	7	F4	21	P3	35	VEOGU	49	R7
	8	F8	22	Pz	36	VEOGL	50	R8
	9	FC5	23	P4	37	L1	51	AccX
	10	FC1	24	P8	38	L2	52	AccY
	11	FC2	25	PO7	39	L4	53	AccZ
	12	FC6	26	PO3	40	L5	54	GyroX
	13	C3	27	POz	41	L6	55	GyroY
	14	Cz	28	PO4	42	L7	56	GyroZ
**Configuration**	Name				Description			
	epo.x				Raw data			
	epo.y				Class labels			
	epo.fs				Sampling frequency			
	epo.t				Time points			
	epo.className				Class name			
	epo.file				File name			
	epo.clab				Channel information			
	mnt.x				X coordinates for channel position			
	mnt.y				Y coordinates for channel position			
	mnt.pos_3d				3D coordinates for channel position			
	mnt.clab				Channel information			

REFERENCES

An, S., Kim, S., Chikontwe, P., and Park, S. H. (2020). Few-shot relation learning with attention for EEG-based motor imagery classification. In *2020 IEEE/RSJ International Conference on Intelligent Robots and Systems (IROS)* (IEEE), 10933–10938

Arad, E., Bartsch, R. P., Kantelhardt, J. W., and Plotnik, M. (2018). Performance-based approach for movement artifact removal from electroencephalographic data recorded during locomotion. *PloS One* 13, e0197153

Azab, A. M., Mihaylova, L., Ang, K. K., and Arvaneh, M. (2019). Weighted transfer learning for improving motor imagery-based brain–computer interface. *IEEE Trans. Neural Syst. Rehabil Eng.* 27, 1352–1359

Chambon, S., Galtier, M. N., Arnal, P. J., Wainrib, G., and Gramfort, A. (2018). A deep learning architecture for temporal sleep stage classification using multivariate and multimodal time series. *IEEE Trans. Neural Syst. Rehabil. Eng.* 26, 758–769

Chuang, C.-H., Chang, K.-Y., Huang, C.-S., and Jung, T.-P. (2021). IC-U-Net: a U-Net-based denoising autoencoder using mixtures of independent components for automatic EEG artifact removal. *arXiv preprint arXiv:2111.10026*

Cooney, C., Korik, A., Folli, R., and Coyle, D. (2020). Evaluation of hyperparameter optimization in machine and deep learning methods for decoding imagined speech EEG. *Sensors* 20, 4629

Fahimi, F., Dosen, S., Ang, K. K., Mrachacz-Kersting, N., and Guan, C. (2020). Generative adversarial networks-based data augmentation for brain-computer interface. *IEEE Trans. Neural Netw. Learn. Syst.*

Freer, D. and Yang, G.-Z. (2020). Data augmentation for self-paced motor imagery classification with C-LSTM. *J. Neural Eng.* 17, 016041

Gao, Z., Wang, X., Yang, Y., Mu, C., Cai, Q., Dang, W., et al. (2019). EEG-based spatio–temporal convolutional neural network for driver fatigue evaluation. *IEEE Trans. Neural Netw. Learn. Syst.* 30, 2755–2763

García-Salinas, J. S., Villaseñor-Pineda, L., Reyes-García, C. A., and Torres-García, A. A. (2019). Transfer learning in imagined speech EEG-based BCIs. *Biomed. Signal Process. Control* 50, 151–157

Kaongoen, N., Choi, J. H., and Jo, S. (2021). Speech-imagery-based BCI system using ear-EEG. *J. Neural Eng.* 18, 016023

Karuppusamy, N. S. and Kang, B.-Y. (2020). Multimodal system to detect driver fatigue using EEG, gyroscope, and image processing. *IEEE Access* 8, 129645–129667

Kilicarslan, A. and Vidal, J. L. C. (2019). Characterization and real-time removal of motion artifacts from EEG signals. *J. Neural Eng.* 16, 056027

Ko, W., Jeon, E., Jeong, S., and Suk, H.-I. (2021). Multi-scale neural network for EEG representation learning in BCI. *IEEE Comput. Intell. Mag.* 16, 31–45

Kostas, D., Aroca-Ouellette, S., and Rudzicz, F. (2021). BENDR: using transformers and a contrastive self-supervised learning task to learn from massive amounts of EEG data. *arXiv preprint arXiv:2101.12037*

Kostas, D. and Rudzicz, F. (2020). Thinker invariance: enabling deep neural networks for BCI across more people. *J. Neural Eng.* 17, 056008

Kristensen, A. B., Subhi, Y., and Puthusserypady, S. (2020). Vocal imagery vs intention: Viability of vocal-based EEG-BCI paradigms. *IEEE Trans. Neural Netw. Learn. Syst.* 28, 1750–1759

Lee, S.-H., Lee, M., and Lee, S.-W. (2020a). Neural decoding of imagined speech and visual imagery as intuitive paradigms for BCI communication. *IEEE Trans. Neural Syst. Rehabil Eng.* 28, 2647–2659

Lee, Y.-E., Kwak, N.-S., and Lee, S.-W. (2020b). A real-time movement artifact removal method for ambulatory brain-computer interfaces. *IEEE Trans. Neural Syst. Rehabil Eng.* 28, 2660–2670

Li, B., Zhang, Z., Duan, F., Yang, Z., Zhao, Q., Sun, Z., et al. (2021a). Component-mixing strategy: A decomposition-based data augmentation algorithm for motor imagery signals. *Neurocomputing* 465, 325–335

Li, F., Chao, W., Li, Y., Fu, B., Ji, Y., Wu, H., et al. (2021b). Decoding imagined speech from EEG signals using hybrid-scale spatial-temporal dilated convolution network. *J. Neural Eng.* 18, 0460c4

Lin, C.-T., Chuang, C.-H., Hung, Y.-C., Fang, C.-N., Wu, D., and Wang, Y.-K. (2020). A driving performance forecasting system based on brain dynamic state analysis using 4-D convolutional neural networks. *IEEE Trans. Cybern.* , 1–9

Luo, Y., Zhu, L.-Z., Wan, Z.-Y., and Lu, B.-L. (2020). Data augmentation for enhancing EEG-based emotion recognition with deep generative models. *J. Neural Eng.* 17, 056021

Malcolm, B. R., Foxe, J. J., Butler, J. S., Mowrey, W. B., Molholm, S., and De Sanctis, P. (2019). Long-term test-retest reliability of event-related potential (ERP) recordings during treadmill walking using the mobile brain/body imaging (MoBI) approach. *Brain Res.* 1716, 62–69

McCartney, B., Martinez-del Rincon, J., Devereux, B., and Murphy, B. (2019). A zero-shot learning approach to the development of brain-computer interfaces for image retrieval. *PloS One* 14, e0214342

Moctezuma, L. A., Torres-García, A. A., Villaseñor-Pineda, L., and Carrillo, M. (2019). Subjects identification using EEG-recorded imagined speech. *Expert Syst. Appl.* 118, 201–208

Mousavi, S., Afghah, F., and Acharya, U. R. (2019). SleepEEGNet: Automated sleep stage scoring with sequence to sequence deep learning approach. *PloS One* 14, e0216456

Nguyen, C. H., Karavas, G. K., and Artemiadis, P. (2017). Inferring imagined speech using EEG signals: a new approach using Riemannian manifold features. *J. Neural Eng.* 15, 016002

Nordin, A. D., Hairston, W. D., and Ferris, D. P. (2019a). Faster gait speeds reduce alpha and beta EEG spectral power from human sensorimotor cortex. *IEEE Trans. Biomed. Eng.* 67, 842–853

Nordin, A. D., Hairston, W. D., and Ferris, D. P. (2019b). Human electrocortical dynamics while stepping over obstacles. *Sci. Rep.* 9, 1–12

Perslev, M., Jensen, M. H., Darkner, S., Jennum, P. J., and Igel, C. (2019). U-time: A fully convolutional network for time series segmentation applied to sleep staging. *arXiv preprint arXiv:1910.11162*

Saha, P. and Fels, S. (2019). Hierarchical deep feature learning for decoding imagined speech from EEG. *Proceedings of the AAAI Conference on Artificial Intelligence (AAAI)* 33, 10019–10020

Shahin, M., Ahmed, B., Hamida, S. T.-B., Mulaffer, F. L., Glos, M., and Penzel, T. (2017). Deep learning and insomnia: Assisting clinicians with their diagnosis. *IEEE J. Biomed. Health Inform.* 21, 1546–1553

Shim, K.-H., Jeong, J.-H., and Lee, S.-W. (2020). Gradual relation network: Decoding intuitive upper extremity movement imaginations based on few-shot EEG learning. *arXiv preprint arXiv:2005.02602*

Singh, A., Lal, S., and Guesgen, H. W. (2019a). Reduce calibration time in motor imagery using spatially regularized symmetric positives-definite matrices based classification. *Sensors* 19, 379

Singh, A., Lal, S., and Guesgen, H. W. (2019b). Small sample motor imagery classification using regularized Riemannian features. *IEEE Access* 7, 46858–46869

Sousa, T., Amaral, C., Andrade, J., Pires, G., Nunes, U. J., and Castelo-Branco, M. (2017). Pure visual imagery as a potential approach to achieve three classes of control for implementation of BCI in non-motor disorders. *J. Neural Eng.* 14, 046026

Sun, W., Su, Y., Wu, X., and Wu, X. (2020). A novel end-to-end 1D-ResCNN model to remove artifact from EEG signals. *Neurocomputing* 404, 108–121

Tian, X., Ding, N., Teng, X., Bai, F., and Poeppel, D. (2018). Imagined speech influences perceived loudness of sound. *Nat. Hum. Behav.* 2, 225–234

Wu, E. Q., Hu, D., Deng, P.-Y., Tang, Z., Cao, Y., Zhang, W.-M., et al. (2020a). Nonparametric Bayesian prior inducing deep network for automatic detection of cognitive status. *IEEE Trans. Cybern.* , 1–14

Wu, E. Q., Zhu, L.-M., Zhang, W.-M., Deng, P.-Y., Jia, B., Chen, S.-D., et al. (2020b). Novel nonlinear approach for real-time fatigue EEG data: An infinitely warped model of weighted permutation entropy. *IEEE Trans. Intell. Transp. Syst.* 1, 2437–2448

Yan, R., Li, F., Zhou, D. D., Ristaniemi, T., and Cong, F. (2020). Automatic sleep scoring: A deep learning architecture for multi-modality time series. *J. Neurosci. Methods* 348, 108971

Yang, L., Song, Y., Ma, K., and Xie, L. (2021). Motor imagery EEG decoding method based on a discriminative feature learning strategy. *IEEE Trans. Neural Syst. Rehabil. Eng.* 29, 368–379

Zhang, D., Yao, L., Chen, K., Wang, S., Haghighi, P. D., and Sullivan, C. (2019). A graph-based hierarchical attention model for movement intention detection from EEG signals. *IEEE Trans. Neural Syst. Rehabil. Eng.* 27, 2247–2253

Zhang, K., Robinson, N., Lee, S.-W., and Guan, C. (2021). Adaptive transfer learning for eeg motor imagery classification with deep convolutional neural network. *Neural Netw.* 136, 1–10

Zhang, R., Zong, Q., Dou, L., Zhao, X., Tang, Y., and Li, Z. (2020). Hybrid deep neural network using transfer learning for EEG motor imagery decoding. *Biomed. Signal Process. Control* 63, 102144
